# Folate-based binuclear Mn(II) chelates with 2,2’-bipyridine/1,10-phenanthroline as targeted anticancer agents for colon cancer cells

**DOI:** 10.1038/s41598-025-12251-9

**Published:** 2025-07-31

**Authors:** Mona S. Ragab, Marwa H. Soliman, Marwa M. Sharaky, Abdelrahman Saad, Mohamed R. Shehata, Mohamed M. Shoukry, Mohamed A. Ragheb

**Affiliations:** 1 Department of Chemistry, Faculty of Science, Cairo University, P.O. 12613, Giza, Egypt; 2 Department of Chemistry (Biochemistry Division), Faculty of Science, Cairo University, P.O. 12613, Giza, Egypt; 3 Pharmacology Unit, Cancer Biology Department, National Cancer Institute, Cairo University, Giza, Egypt; 4 Biotechnology Department, Faculty of Nanotechnology for Postgraduate Studies, Cairo University, Sheikh Zayed Branch Campus, P.O. 12588, Giza, Egypt; 5 Faculty of Biotechnology, October University for Modern Sciences and Arts, Giza, Egypt

**Keywords:** New binuclear Mn(II) chelates, DFT computations, Anti-apoptotic proteins, Positive folate receptor, Cytotoxicity, And HCT116 cell arrest at S- phase., Drug development, Bioinorganic chemistry, Cancer

## Abstract

Researchers have shown significant interest in the chemistry of mixed ligand transition metal chelates due to their diverse applications and bonding features. Herein, we have reported the synthesis of two new binuclear Mn(II) chelates with the composition of [Mn_2_(FA)(Bpy)_2_(H_2_O)_2_Cl_2_].7H_2_O (Chelate 1) and [Mn_2_(FA)(Phen)_2_(H_2_O)_2_Cl_2_].7H_2_O (Chelate 2) (where, FA = folate anion, Bpy = 2,2’-bipyridine, Phen = 1,10-phenanthroline). The folate anion acts as a bridging ligand to obtain the binuclear complexes with the suggested composition. The chelates have been characterized by using elemental analysis, spectroscopic (FTIR, UV-vis), EI-MS and TGA techniques. Further, the results from density functional theory (DFT) computations support well the suggested structures, which indicate the development of distorted octahedral geometries around each Mn(II) center for both binuclear complexes. The sulforhodamine B (SRB) assay was used to assess the cytotoxicity of the chelates against a series of folate receptor-positive and -negative cell lines. The prepared chelates exhibit significant cytotoxic effects against folate receptor-positive cell lines, with colon cancer HCT116 cells being the most affected. Furthermore, the anticancer mechanism of action on the HCT116 cell line was investigated by analyzing the wound healing, cell cycle profile, expression of pro-apoptotic and anti-apoptotic proteins, as well as examining the morphological changes in treated cells. The results provided compelling evidence of binuclear Mn(II) chelates-induced cellular and nuclear alterations.

**Supplementary Information:**

The online version contains supplementary material available at 10.1038/s41598-025-12251-9.

## Introduction

Vitamin B9, frequently known as folic acid, is vital for human health from a group of vitamins called water-soluble vitamins. Its chemical structure includes pteroic acid and glutamic acid joined together by an amide bond, leading to its versatility in its ligational aspects. The glutamate moiety’s γ-carboxyl groups can act as a monodentate ligand, a bridging bidentate ligand coordinating with two metals, or bidentate ligand binding to a single metal; all three distinct coordination modes were documented^1^. Folic acid plays a key role in producing new cells and is essential for synthesizing and maintaining DNA and RNA^2^Its deficiency leads to DNA hypomethylation^3^DNA strand breaks^4^and abnormal gene expression^3^. Moreover, it is crucial in the synthesis of the purine ring and the conversion of 2-deoxyuridine monophosphate to thymidine monophosphate via S-adenosylmethionine, along with other essential substrates and cofactors. Therefore, it is not unexpected that abnormally low folate levels are associated with a range of developmental anomalies and diseases, including Alzheimer’s dementia, neural disorders, inflammatory disease, pregnancy complications, cancer, and coronary artery disease. Severe deficiencies cause noticeable symptoms, like anemia in folate deficiency^5^. To date, folate-based conjugates of potential anticancer effects^6,7^have been synthesized and effectively evaluated in cancer cells that overexpress the folate receptor (FR) on their surfaces. However, there is a gap in literature concerning the coordination chemistry of metal-folate complexes and even less information about their chemotherapeutic potencies.

Interestingly, manganese(II) complexes have shown promise as effective cytotoxic agents in addition to their DNA binding and cleavage propensity^8–12^. As well, manganese ions are essential for diverse significant biochemical processes such as regulation of blood glucose levels, immunity functions, cellular energy, growth of bones, cholesterol biosynthesis, and coagulation of blood^13^. It also plays a significant role in the body as an antioxidant against reactive oxygen species (ROS) to reduce oxidative stress within the human body^14^. Intriguingly, the particular attention received by the manganese is mainly due to the presence of different oxidation states (Mn^2+^, Mn^3+^, and Mn^4+^). Among these, Mn^2+^ ions exhibit the highest stability compared to Mn^3+^ and Mn^4+^ ions. Numerous mono- and binuclear Mn(III/II) complexes with catalase activity, primarily utilizing Schiff base and other various ligands have been reported in the literature in an attempt to imitate the enzymes’ active sites^15–17^. In summary, the biological activity studies of manganese complexes have emerged as an attractive research field in modern bioinorganic chemistry. The cellular action mode of such manganese-based inorganic chelates mainly depends on ROS generation, leading to oxidative stress in various cancer cell lines as reported by Ansari et al.^18,19^. Furthermore, it has been reported that Mn(II) compounds demonstrate similar or higher cytotoxic effects on cell lines such as breast cancer (MCF-7), human lung adenocarcinoma (A549) compared to Cu(II) and Fe(III) compounds with phenanthroline and pyridine derivatives^20^. It has been previously reported that three manganese(II) chelates are undergoing clinical trials: EUK-134, an antioxidant evaluated for the treatment of stroke^21–23^; M40403, an antioxidant investigated for Parkinson’s disease and as a radioprotective agent^24–26^; and AEOL-10,150, a subcutaneous agent studied for conditions including spinal cord injury, amyotrophic lateral sclerosis, stroke, pneumonia, and mucositis^27–29^. However, many cytotoxic manganese chelates do not differentiate between normal and cancer cells, resulting in damage to normal cells. Improving the specificity of cytotoxic agents through targeted mechanisms can help minimize damage to healthy tissue. Recent reports suggest that targeting cancer cells can be achieved by combining metal complexes with antibodies, proteins, or peptides^30,31^. Additionally, studies have shown that the folate receptor is overexpressed on the surfaces of various cancer cells, such as ovarian (A2780), breast (MDA-MB-231), colon (Caco-2), cervical (HeLa-IU1), and nasopharyngeal (KB)^32–35^compared to normal cells. So, the compounds including a covalently linked folic acid could successfully reach the targeted cancer cells and incorporate through a non-destructive receptor-mediated endocytic pathway^36^. As a result, metal chelates based on folic acid have attracted significant interest as a potential approach to target tumor cells selectively^37^. On the other hand, the investigation of 2,2′-bipyridine/1,10-phenanthroline-based manganese(II) chelates has attracted great attention in recent years^38^. These ligands are bidentate N-donor heterocyclic compounds that efficiently form stable coordination compounds with various transition metals due to their chelating nature^39^. As a result, they are widely used as ancillary ligands in coordination chemistry. Their electron-deficient aromatic systems make them excellent electron acceptors, allowing them to stabilize metal chelates through various unconventional non-covalent interactions^40^. Many chelates containing such compounds demonstrate cytotoxic activity against tumor cells, likely through mechanisms such as DNA binding and/or cleavage, mitochondrial damage, reactive oxygen species generation, or topoisomerase inhibition^41,42^.

The present work aims to demonstrate the interaction between Mn(II) metal ions and folic acid in the presence of co-ligands such as 2,2’-bipyridine or 1,10-phenanthroline. The two binuclear mixed ligands Mn(II) chelates, [Mn_2_(FA)(Bpy/Phen)_2_(H_2_O)_2_Cl_2_]·7H_2_O, were prepared and identified using different spectroscopic and non-spectroscopic tools. The cytotoxic behavior of the two chelates was assessed against HeLa, HCT116, CaCo-2, MCF-7, and A549 cancer cells. The chelates exhibited an exceptional cytotoxic response toward the cancer cells studied. Remarkably, the chelates exhibited the highest cytotoxic activity against HCT116 cancer cells with IC_50_ = 5.8 µg/ml for Chelate 1 and 7.2 µg/ml for Chelate 2. The binuclear manganese systems can significantly impact the proliferation of both cancer and normal cells, unveiling the effects of these chelates on the cell cycle and other cellular functions in HCT116 cells. The ability of how well the chelates could bind to DNA was monitored through UV-vis absorption spectroscopy. The plasmid DNA cleavage pattern of chelates without any additives was examined using agarose gel electrophoresis.

## Experimental

### Materials and instrumentation

All chemicals and solvents used in this study were of the highest purity and were sourced from Sigma Aldrich. We used a Shimadzu UV 1800 spectrophotometer to obtain electronic spectra and FT-IR 460 Plus spectrophotometer to capture the Fourier transform KBr disc infrared (FTIR) spectra of the chelates. A Vario EL III (CHN) analyzer was employed to determine the C, H, and N% content. The EI-MS of the chelates was recorded at 70 eV with the aid of a SHIMADZU QP-2010 plus mass spectrometer. The molar conductance of the substances in DMF (10^−3^ M) was determined using a Jenco conductivity meter 3173. Thermal gravimetric analysis was collected using Shimadzu’s TG-60 H thermal analyzer with a dynamic N_2_ flow of 20 ml/min. To determine the magnetic susceptibilities of the chelates, a Sherwood scientific magnetic balance and a Gouy balance were used, with Hg[Co(CSN)_4_] as the calibrant. Measurements were taken using freshly prepared solutions at room temperature.

### Computational methods

To determine the molecular conformation of the complexes without relying on a single crystal X-ray structure assessment, DFT optimization was conducted using the Gaussian09 software^43^. Becke3-Lee-Yang-Parr (B3LYP) level of theory combined with 6-311G++(d, p) and Los Alamos National Laboratory 2 Double-Zeta (LanL2DZ) basis sets were used to simulate the molecular geometry of the synthesized metal chelates. Visualization was assessed using the Gauss View 5.0 program.

### Preparation of [Mn_2_(FA)(Bpy/Phen)_2_(H_2_O)_2_Cl_2_]. 7H_2_O (1) and (2)

**Scheme 1:**
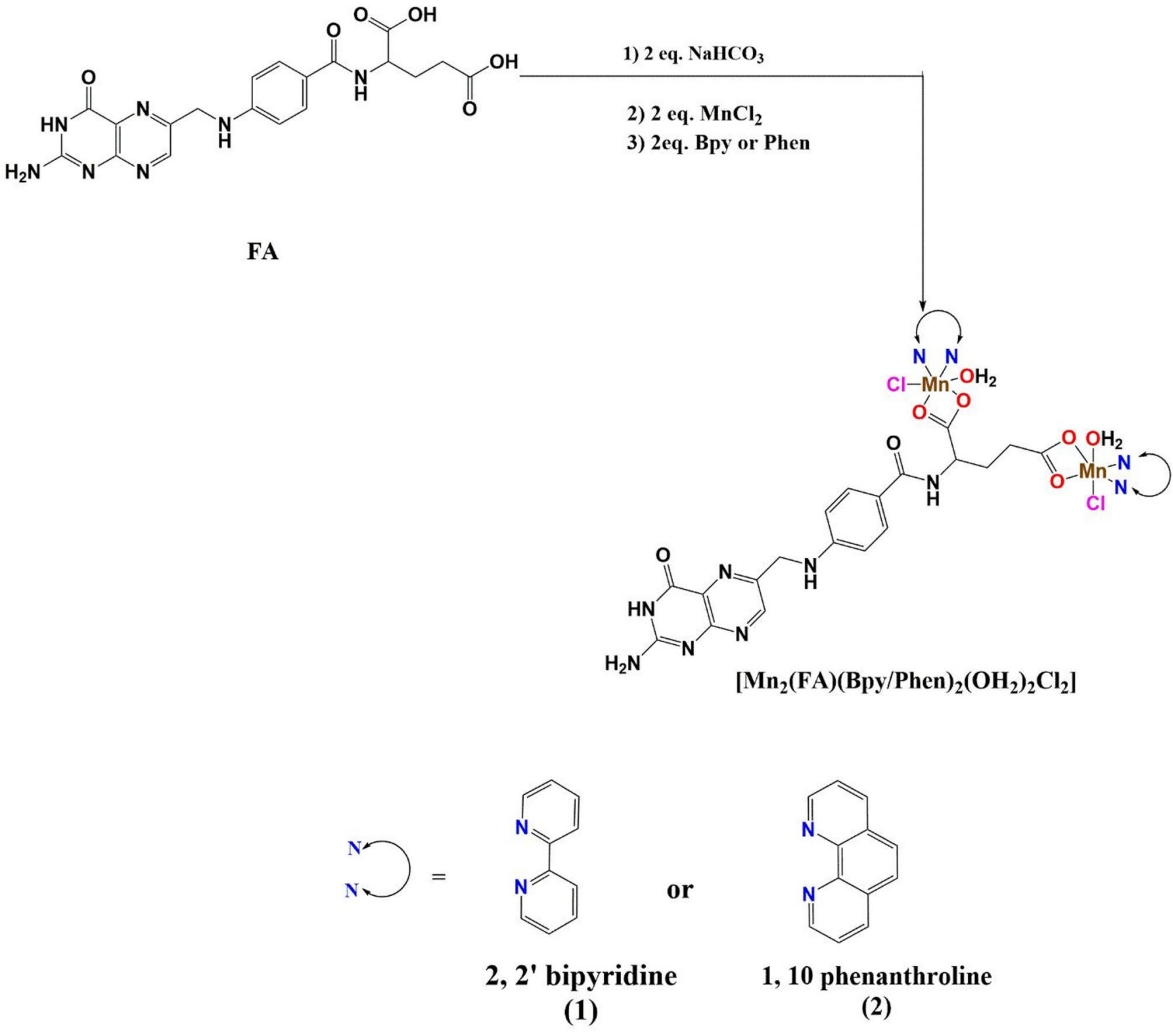
Schematic route of the preparation of Chelates 1 and 2.

As presented in Scheme 1, 1 mmol of folic acid (0.441 g) was neutralized by 2 mmol of sodium bicarbonate (0.168 g) with a small amount of distilled water and stirred till reaching a completely clear yellow solution. Then, the yellow solution was mixed with 2 mmol of MnCl_2_.2H_2_O (0.396 g) and stirred for 10 min till yellow ppt was formed. The yellow ppt was mixed with 2 mmol of methanolic solution of 2,2’-bipyridine (Bpy) (0.312 g) (Chelate 1) or 1,10-phenanthroline (Phen) (0.396 g) (Chelate 2) and refluxed for 3 h till the formation of dark brown ppt. The resulting binuclear Mn(II) complexes were filtered and left to dry.

- The yield of Chelate 1: 95%, M.wt = 1094.57 g/mol, m.p.> 250°C. Anal. Found: C, 42.70; H, 4.15; N, 14.02; Calc. for C_39_H_51_Cl_2_Mn_2_N_11_O_15_ (1094.68 g/mol): C, 42.79; H, 4.70; N, 14.08. IR (KBr, cm^−1^): ν(H_2_O) 3672, ν(NH_2_) 3363, ν(NH) 3278, (ν(C = O)) _COO_- 1743, ν_as_(COO^−^) 1566, (C = C) 1520, ν_s_(COO^−^) 1381, ν(CN) 1265. Λ_m_ (10^− 3^ M in DMF, Ω^-1^cm^2^mol^−1^) = 18. EI- MS [m/z]: 1096.93 (M + 2).
- The yield of Chelate 2: 93%, M.wt = 1142 g/mol, m.p.> 250°C. Anal. Found: C, 42.00; H, 4.23; N, 13.15; Calc. for C_43_H_51_Cl_2_Mn_2_N_11_O_15_ (1142.72 g/mol): C, 42.20 H, 4.50; N, 13.48. IR (KBr, cm-1): ν(H_2_O) 3672, ν(NH_2_) 3363, ν(NH) 3278, (ν(C = O)) _COO_- 1735, ν_as_(COO^−^) 1573, δ(C = C) 1520, ν_s_(COO^−^) 1388, ν(CN) 1265. Λ_m_ (10^− 3^ M in DMF, Ω^- 1^ cm^2^ mol^-1^) = 8. EI- MS [m/z]: 996.00 (M-2-8H_2_O).

### DNA interaction assays

#### DNA binding

The two chelates are poorly soluble in water but soluble in 5% DMSO. The used DNA was calf thymus DNA (CT-DNA), which is dissolved in Tris–NaCl (5 mM Tris–HCl/50 mM NaCl (pH 7.1)) buffer^44^. The DNA purity was more than 1.8 after it was tested by measuring the UV absorbance ratio value of (A_260_/A_280_). The absorbance value at 260 nm (ε = 6600 M^−1^ cm^−1^) was used to calculate the amount of DNA per nucleotide^44–46^. The concentration used of the two chelates [Mn_2_(FA)(Bpy)_2_(H_2_O)_2_Cl_2_]·7H_2_O (1) and [Mn_2_(FA)(Phen)_2_(H_2_O)_2_Cl_2_]·7H_2_O (2), were 60 µM and 30 µM, respectively, in 5% DMSO, followed by addition of CT-DNA with increasing amount over range 0-840 nM for Chelate 1 and 0-220 nM for Chelate 2. The reaction was done at room temperature, and the incubation time of the components of the reaction mixture was 5 min before the absorption spectrum was recorded to allow for achieving the state of equilibrium^44^. The absorbance (A) was measured in a Shimadzu UV–Vis spectrophotometer (Japan) with a 1 cm cuvette. By using Wolfe–Shimer Eq. (1), the DNA binding constant K_b_ calculations were done, where [DNA] is the concentration of CT-DNA, ε_a_ = A_obs_ / [compound], ε_f_ = is the extinction coefficient of the chelates remaining free in solution and ε_b_ = is the extinction coefficient of the chelates when fully bound to DNA^44^.

1 $$\left[ {DNA} \right]/({\varepsilon _{\text{a}}} - {\varepsilon _{\text{f}}})=\left[ {DNA} \right]/{\text{ }}({\varepsilon _{\text{b}}} - {\varepsilon _{\text{f}}})+1/[{K_{\text{b}}}({\varepsilon _{\text{b}}} - {\varepsilon _{\text{f}}})].$$

#### DNA cleavage propensity

The DNA cleavage activity was assessed *via* agarose gel electrophoresis. Approximately 0.4 µg of pBR322 plasmid, dissolved in 5 mM Tris buffer (pH 7.1), was treated with 100 µM of each Mn(II) chelate, [Mn_2_(FA)(Bpy)_2_(H_2_O)_2_Cl_2_].7H_2_O (Chelate 1) and [Mn_2_(FA)(Phen)_2_(H_2_O)_2_Cl_2_].7H_2_O (Chelate 2), at 37°C, followed by incubation for 2.5 h. After incubation, 1% agarose gel electrophoresis was conducted for 2 h at 70 V in a Tris-boric acid-EDTA buffer. The DNA bands were then visualized under UV light, and images were captured. The degree of DNA cleavage was quantified using ImageJ software^44,47^. By measuring the transformation of pBR322 plasmid from its supercoiled (SC) form to nicked (NC) and linear (L) forms, enabling an assessment of the nuclease activity of the tested compounds^48^.

### Biological assays

#### Cell culture

Human carcinoma cell lines, colorectal carcinoma (HCT116), cervical cancer (HeLa), colorectal adenocarcinoma (Caco-2), breast cancer (MCF-7), and lung carcinoma (A549), in addition to normal human skin fibroblasts (HSF) cell line were obtained from the American Type Culture Collection (ATCC, Minnesota, USA) and were maintained in DMEM supplemented with 10% fetal bovine serum and 1% penicillin-streptomycin, and incubated in 5% CO_2_ in a humidified atmosphere at 37°C.

#### *In vitro* cytotoxicity assay

The cytotoxic activities of Chelate 1 [Mn_2_(FA)(Bpy)_2_(H_2_O)_2_Cl_2_]·7H_2_O and Chelate 2 [Mn_2_(FA)(Phen)_2_(H_2_O)_2_Cl_2_]·7H_2_O were assessed as on different positive and negative folate receptor cell lines using the Sulphorhodamine-B (SRB) assay^49^. In 96-well microtiter plates, cells were seeded at a density of 3 × 10^3^ cells/well. They were allowed to adhere for a full day before starting 48 h incubation with either a single dose (100 µg/ml) or graded concentrations (0, 6.25, 12.5, 25, and 50 µg/ml) for IC_50_ determination in the investigated cell lines. After the incubation period, cells were stained with 0.4% SRB dye and fixed with 20% trichloroacetic acid. Spectrophotometric measurements of each well’s optical density (O.D.) were made using an ELISA microplate reader (TECAN SunriseTM, Germany) at 570 nm. The cell survival percentage was calculated as follows:

$${\text{Survival}}\;{\text{ fraction}}={\text{O}}{\text{.D}}.\;\left( {{\text{treated}}\;{\text{cells}}} \right)/{\text{O}}{\text{.D}}{\text{.}}\;\left( {{\text{control}}\;{\text{cells}}} \right)$$

The IC_50_ (concentration that produces 50% of cell growth inhibition) value of each chelate was calculated using sigmoidal dose-response curve-fitting models (Graph Pad Prizm software, version 8.2).

#### Wound healing

HCT116 cells (the most chelate-responsive cells) were cultured at a density of 1 × 10^5^ cells/well in 6-well tissue culture plates at 37°C and 5% CO_2_. After the cells reached a 70–80% confluent monolayer, a 20 µl pipette tip scrape was applied in a straight line to the monolayer to create a cell-free region. To get rid of cell debris, PBS was used twice to rinse the cells. After that, the prepared chelates were immediately put into wells along with their IC_50_ and maintained in the previously stated circumstances to allow cell migration into the medium. After 0 and 48 h, the rate of wound healing was observed using an inverted microscope (Olympus Optical Co., Ltd., Tokyo, Japan) in contrast to control cells. Olympus software was used to assess the wound gap area to get its average distance^50^. Lastly, the percentage of wound closure (Wound closure%) was calculated using the following formula:

$${\text{The}}\;{\text{closure}}\% \;{\text{of}}\;{\text{ the}}\;{\text{wound}}=\left( {{{\text{A}}_{0{\text{h}}}}-{\text{ }}{{\text{A}}_{{\text{xh}}}}} \right)/{{\text{A}}_{0{\text{h}}}} \times 100$$

where, A_0h_ is a closure area of the wound at 0 h, while A_xh_ is at 48 h^51^.

#### Colony formation assay

After the cells had reached around 80% confluency, they were exposed to the IC_50_ concentration of each chelate for a whole day. Cells were trypsinized after 48 h, then plated at a density of roughly 1 × 10^3^ in each well of a 6-well plate and left to develop for another 14 days. Following a PBS wash, the cells were incubated for 30 min in a 0.5% crystal violet solution that contained 3.7% formaldehyde. After that, the crystal violet was removed from the plates using running tap water, and they were left to dry at room temperature; then, images were captured by a digital camera. Finally, the ImageJ program was used to count the colonies^52^.

#### Cell morphology investigation

After plating the cells in 25 cm^2^ tissue culture flasks, they were treated with the synthesized Chelates 1 and 2 at their respective IC_50_ concentrations for 48 h^53^. Images were captured using an inverted microscope (Olympus Optical Co., Ltd., Tokyo, Japan) and compared to those of untreated cells.

#### Cell cycle

After seeding HCT116 cells (5 × 10^4^ cells/well) into 6-well plates and allowing them to grow overnight, the cells were exposed to the IC_50_ concentrations of the produced Chelates 1 and 2. Following their collection and washing, the cells were fixed with ice-cold ethanol and suspended in flow cytometry staining buffer before being treated with the propidium iodide (PI) reagent (Beckman Coulter). Cytexpert software was used for data analysis^54, ^after the samples were evaluated using flow cytometry.

#### Enzyme-linked immunosorbent assay

ELISA kits with catalog numbers ab231929 (Abcam), MBS8804448, MBS2510990 (MyBiosource), SEA626Hu, SEB343Hu, and SEA778Hu (Cloud-Clone Corp) were used to measure the levels of Cyclin E, Cyclin A, CDK2, Caspase-3, BAX, and BCL2 activities, respectively. The ELISA plates contained designated wells for the standard, sample, and blank; the standard has seven wells, while the blank has one. The corresponding wells were filled with each dilution of the standard, samples, or blank. After sealing and incubating the plate, detection reagent A was added, followed by another incubation period. Following aspiration of the solution, the plate was cleaned, detection reagent B was applied, and then additional washing and incubation procedures were carried out. After adding the substrate solution, the plate was incubated and shielded from light. A microplate reader was used to measure the absorbance at 450 nm after a stop solution was introduced. The experiment involved three sample conditions: untreated cells, Chelate 1-treated cells, and Chelate 2-treated cells. For sample preparation, treated and untreated cells were gently washed with cold PBS, and then trypsin was added to facilitate their detachment. The cell pellet was collected by centrifuging them at 1,000×g for 5 min. The cell pellet is then resuspended in fresh lysis buffer and incubated. If required, the cells may also be ultrasonically agitated until the solution became clear. Cell extracts were then collected by centrifugation.

## Results and discussion

### Infrared spectra of the metal chelates

**Fig. 1:**
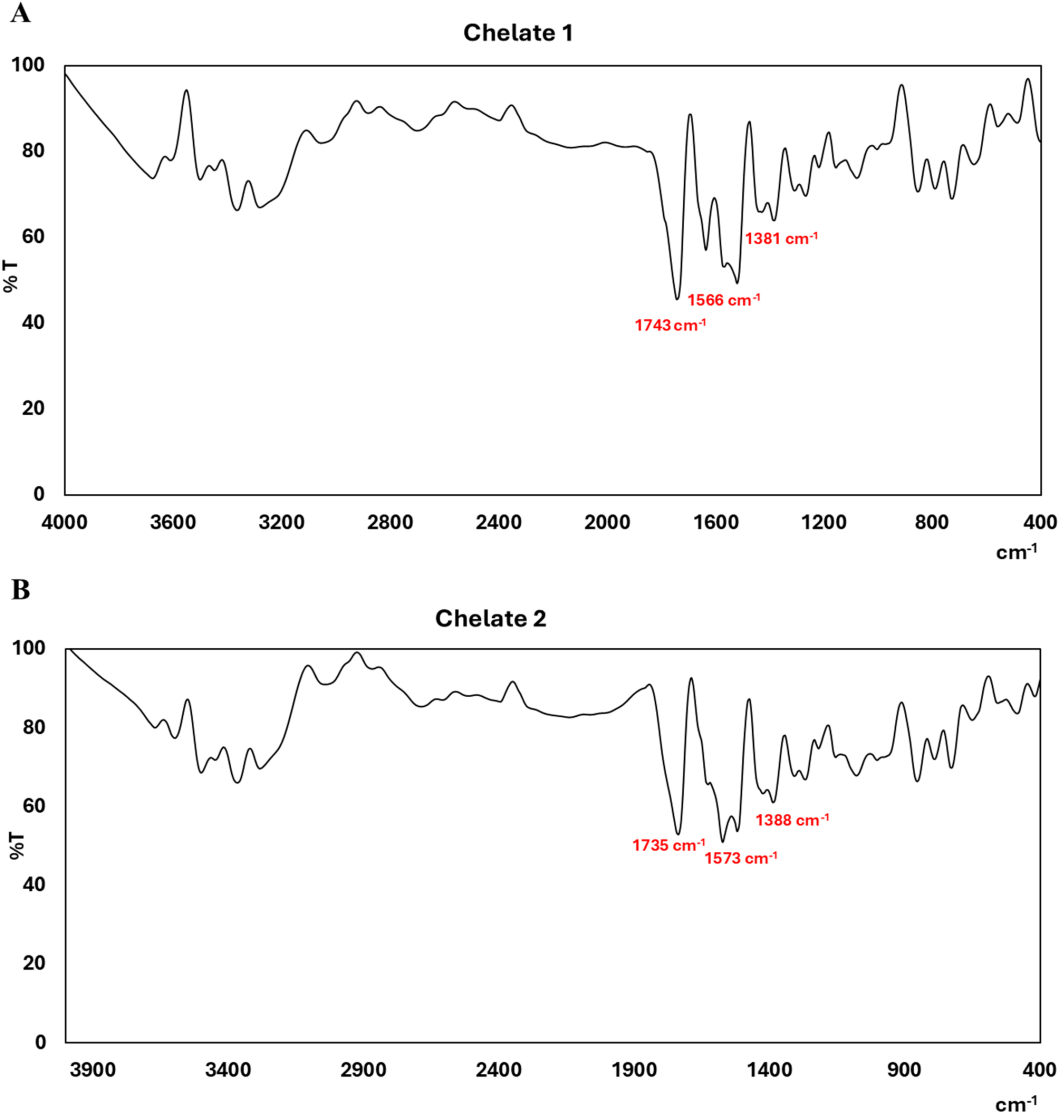
IR charts for the synthesized chelates [Mn_2_(FA)(Bpy)_2_(H_2_O)_2_Cl_2_].7H_2_O (**Chelate 1**; **A**) and [Mn_2_(FA)(Phen)_2_(H_2_O)_2_Cl_2_].7H_2_O (**Chelate 2**; **B**).

In this study, we compare the FTIR spectrum of free folic acid with that of the ternary mixed Chelates **1** and **2**. FTIR is a very useful technique for comprehending the intermolecular interaction of folic acid before and after coordination^55^. As reported by Abd El-Wahed et al.^56^the folic acid spectrum shows a powerful absorption band at 1694 cm^−1^, which was attributed to the stretching vibration of the free ketonic carbonyl group (ν(C = O)) of the carboxylic group. Upon chelation, as presented in IR spectra of chelates, (Fig. 1), this band is shifted to 1743 and 1735 cm^−1^ for Chelate 1 and Chelate 2, respectively, indicating interaction with the metal centers. Interestingly, the band located at 1566, and 1573 cm^−1^ are mainly attributed to the asymmetric ν_as_(COO^−^) stretching vibration while the bands at 1381 and 1388 cm^−1^ for Chelate 1 and Chelate 2, respectively, are mainly attributed to *ν*_*s*_(COO)^−^. Intriguingly, the direction of shift in frequency of the ν_as_(COO^−^) and the ν_s_(COO^−^) bands depends on the coordination mode of the carboxylate group with the metal center. As reported by Nakamoto and McCarthy^57^the carboxylate coordinates in a monodentate manner if the frequencies of ν_as_ (COO^−^) and ν_s_ (COO^−^) were shifted in opposite directions. Herein, the bidentate or bridged bidentate manners were recommended, as the frequencies were shifted in the same direction as the bond orders of both C = O bonds would change equally with Δν = ν_as_ (COO^−^) – ν_s_ (COO^−^) for both chelates is 185 cm^-1^, suggesting that the carboxylate group is symmetrically bonded to the metal center and consisting with the bidentate carboxylate coordination mode^1,58^.

### UV-vis spectra and magnetic moments

**Fig. 2:**
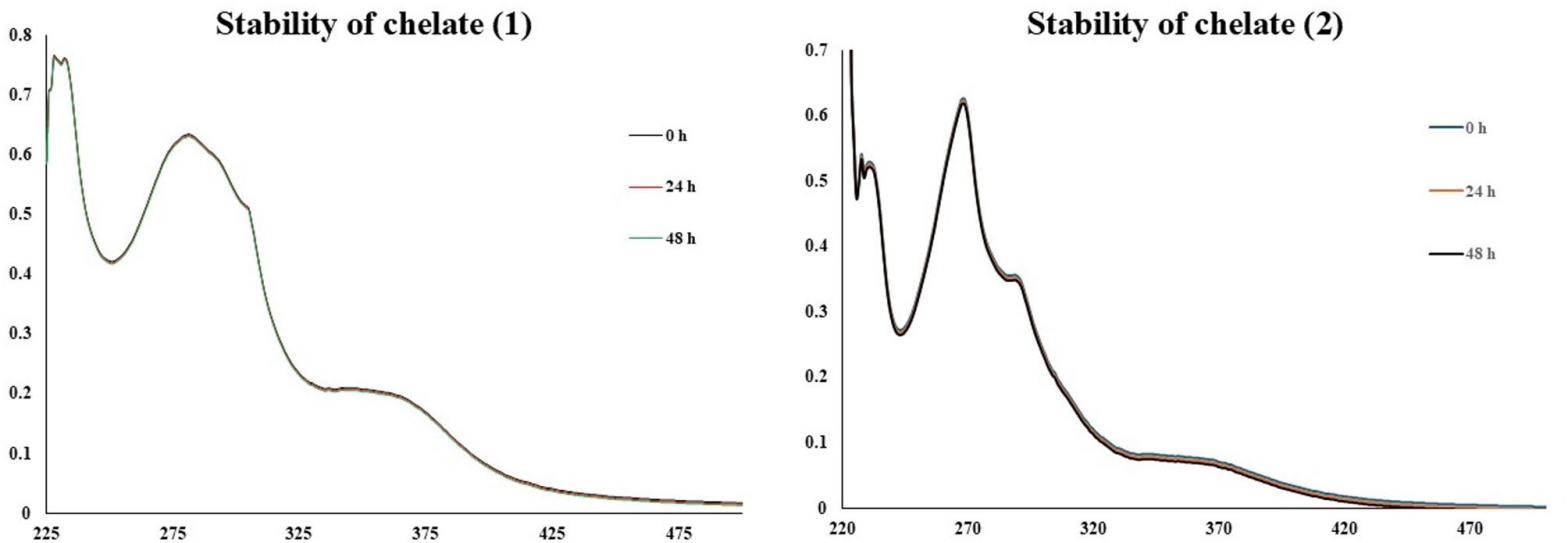
The spectroscopic analysis of Chelates 1 (left) and 2 (right) over 0, 24, and 48 h demonstrates their stability.

In the UV–Vis spectra, the low intensities of d-d transition bands are due to the doubly forbidden transitions from the ^6^A_1g_ state to higher energy states^59^. So, UV–Vis spectra of both Chelates (**1** and **2**) exhibit only the intense absorption bands below 400 nm in DMSO. The spectra manifest the typical π-π* transition at 281 nm and 270 nm for Chelates 1 and 2, respectively^60^. Additionally, the shoulder band detected around 360 nm is assignable to the ligand-to-metal charge transfer (LMCT)^1,61,62^. The spectroscopic inspection over 0, 24, and 48 h suggests that both chelates are sufficiently stable (as shown in Fig. 2).

### Mass spectra

**Fig. 3:**
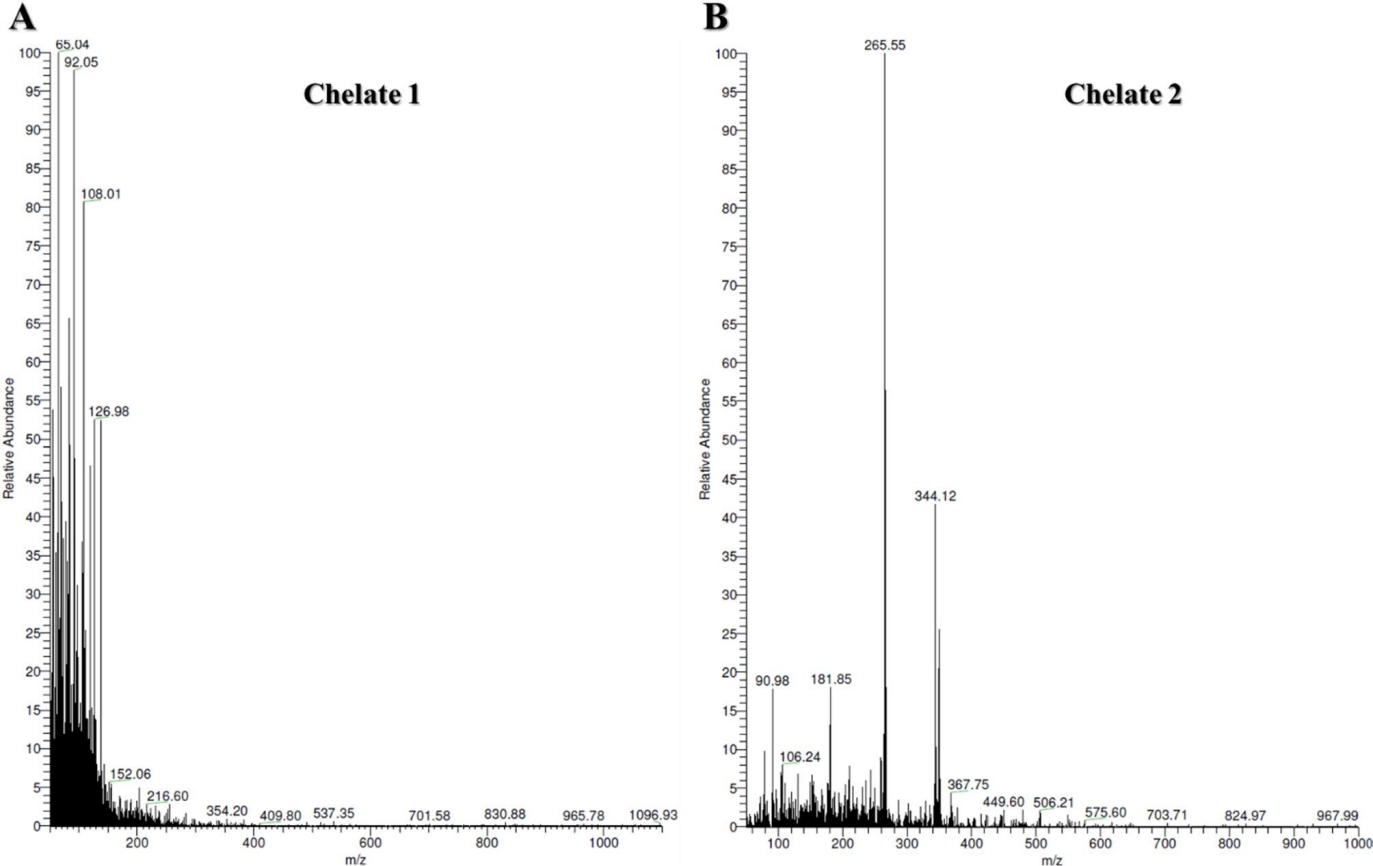
Electron ionization mass spectra of **(A)** [Mn_2_(FA)(Bpy)_2_(H_2_O)_2_Cl_2_]0.7 H_2_O **(Chelate 1**) and **(B)** [Mn_2_(FA)(Phen)_2_(H_2_O)_2_Cl_2_]0.7 H_2_O **(Chelate 2)** recorded in positive ion mode.

The elemental composition and structural integrity of the binuclear manganese complexes were supported by EI mass spectrometry, as shown in Fig. 3. For Chelate 1, [Mn_2_(FA)(Bpy)_2_(H_2_O)_2_Cl_2_]·7H_2_O (M.Wt = 1094.57 g/mol), the mass spectrum shows a peak at m/z 1096.93, corresponding to [M + 2]^+^, which arises from the isotopic distribution of chlorine, specifically the presence of the heavier Cl isotope leading to an M + 2 peak. For Chelate 2, [Mn_2_(FA)(Phen)_2_(H_2_O)_2_Cl_2_]·7H_2_O (M.Wt = 1142 g/mol), the spectrum displays a molecular ion peak at m/z 996.00, which corresponds to the loss of eight water molecules (8 × 18 = 144 Da) and two hydrogen atoms (–2 Da), yielding [M – 8 H_2_O – 2 H]^+^. This indicates a dehydrated and partially fragmented species that retains a single positive charge. All spectra were recorded in positive-ion mode, and these findings align well with the elemental analysis (CHN), supporting the proposed molecular formulations.

### Thermogravimetric analysis (TGA)

**Fig. 4:**
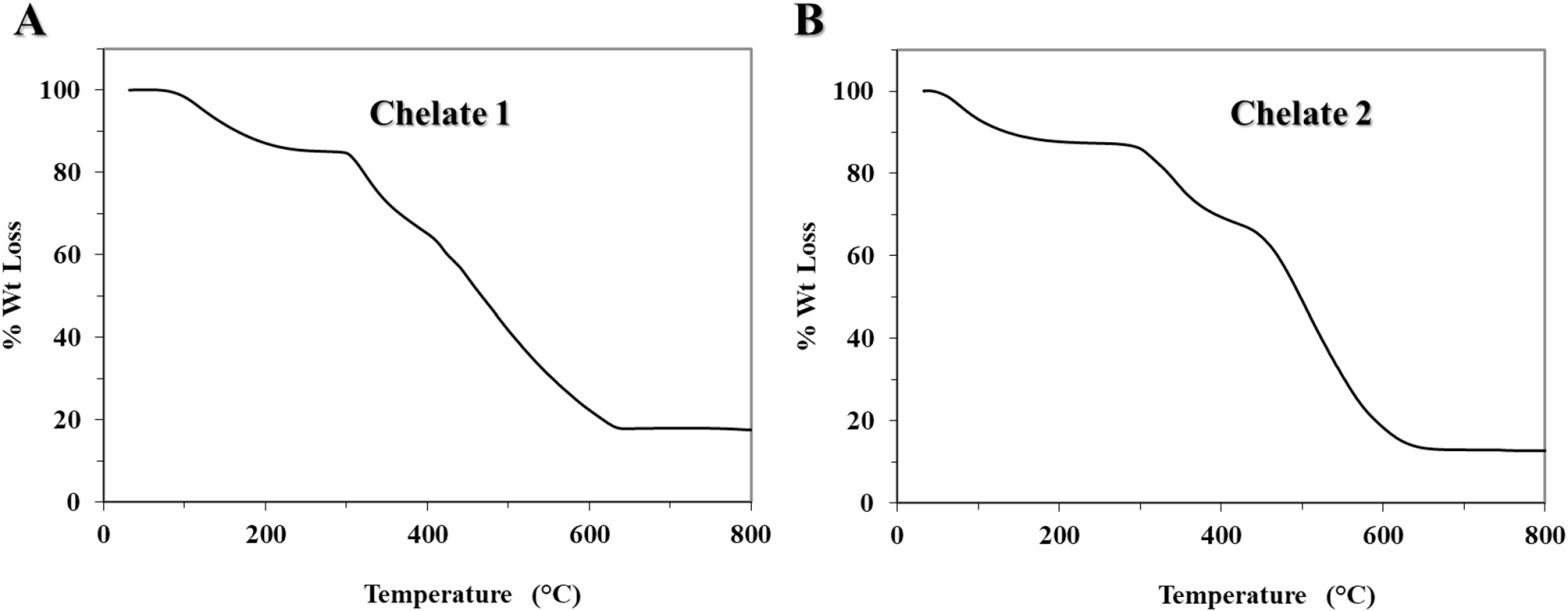
Thermograms of **Chelate 1,** [Mn_2_(FA)(Bpy)_2_(H_2_O)_2_Cl_2_].7H_2_O (**A**), and **Chelate 2,** [Mn_2_(FA)(Phen)_2_(H_2_O)_2_Cl_2_].7H_2_O (**B**).

The TGA thermograms of [Mn_2_(FA)(Bpy)_2_(H_2_O)_2_Cl_2_].7H_2_O (Chelate 1) and [Mn_2_(FA)(Phen)_2_(H_2_O)_2_Cl_2_].7H_2_O (Chelate 2) were displayed in Fig. 4A and 4B, respectively. As seen in the thermogram of Chelate 1, the evaporation of coordinated and uncoordinated water molecules caused an initial small mass loss of 14.80% (calc.=14.80%) up to 236°C. From 236 to 800°C, a noticeable mass loss was noticed in the thermogram, which could reflect the degradation of stable moieties from the complex. In the 236–440°C range, the degradation corresponds to the elimination of two bipyridyl moieties with a mass loss of 28.3% (calc.= 28.5%). From 440 to 465°C, mass loss is continually increased, which may be explained by eliminating two chloride atoms with a mass loss of 6.4% (calc.= 6.5%). The last decomposition step in the range 465–665°C corresponds to the loss of the rest of the folic acid (FA-2CO_2_), with a mass loss of 32.8% (calc.= 32.2%), leaving Mn_2_O_5_ as a stable final residue. The thermal behavior of Chelate 2 is different as displayed in Fig. 4B, in which the evaporation of coordinated and uncoordinated water molecules caused an initial small mass loss of 13.2% (calc.=14.80%) up to 283°C. From 283 to 437°C, the degradation corresponds to the elimination of (2 Chloride atoms + Phen) moieties with mass loss of 20.5% (calc.= 21.9%). From 437 to 800°C, there is a significant increase in mass loss corresponding to the elimination of (FA + Phen), with mass loss of 54.0% (calc.= 53.7%), leaving 2MnO molecules as stable final residue.

### DFT computations

**Fig. 5:**
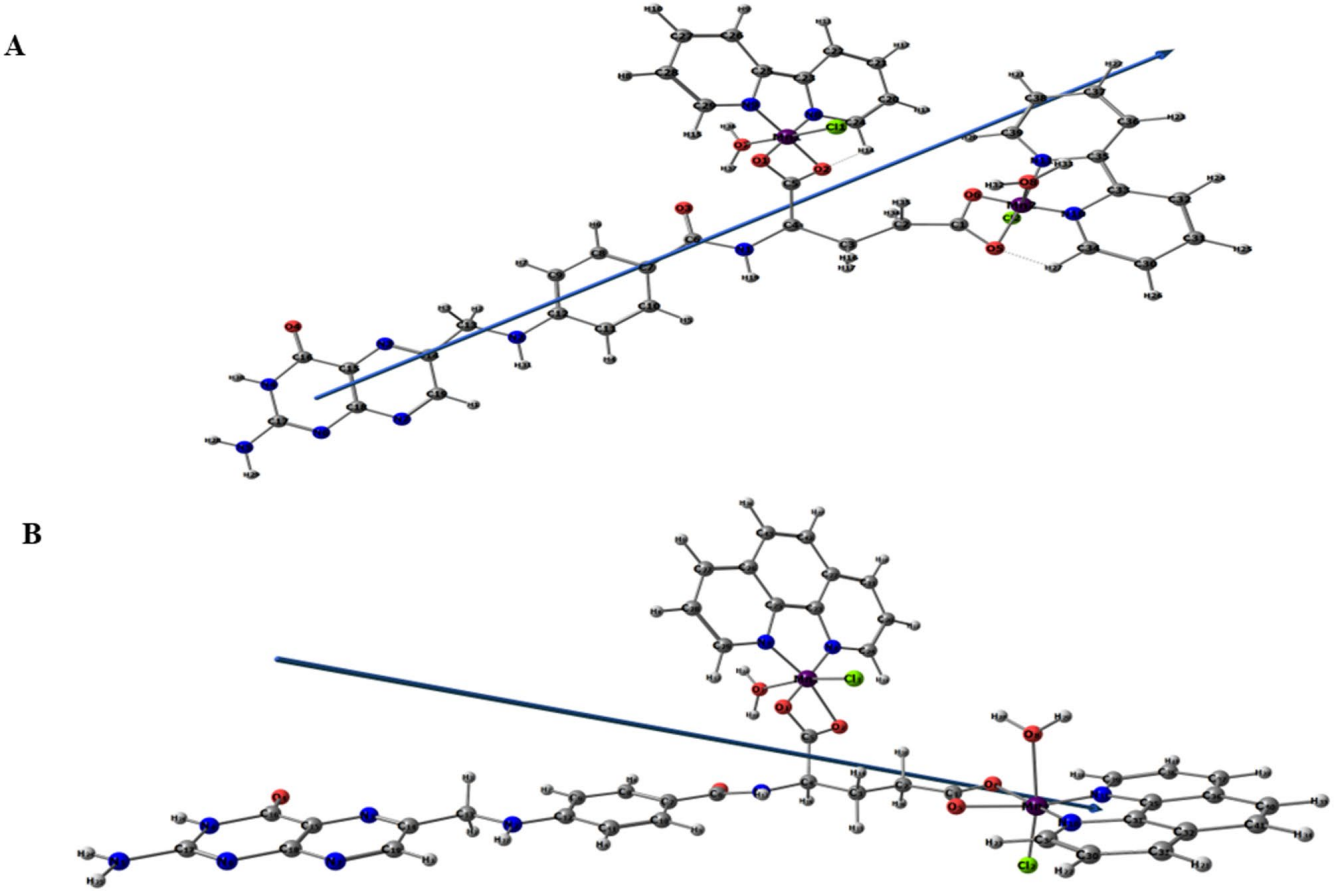
B3LYP/6–311G++(d, p)-LANL2DZ level optimized geometry, vector of dipole moment, and numbering method for the investigated **Chelate 1** (**A**) and **Chelate 2** (**B**).

**Fig. 6:**
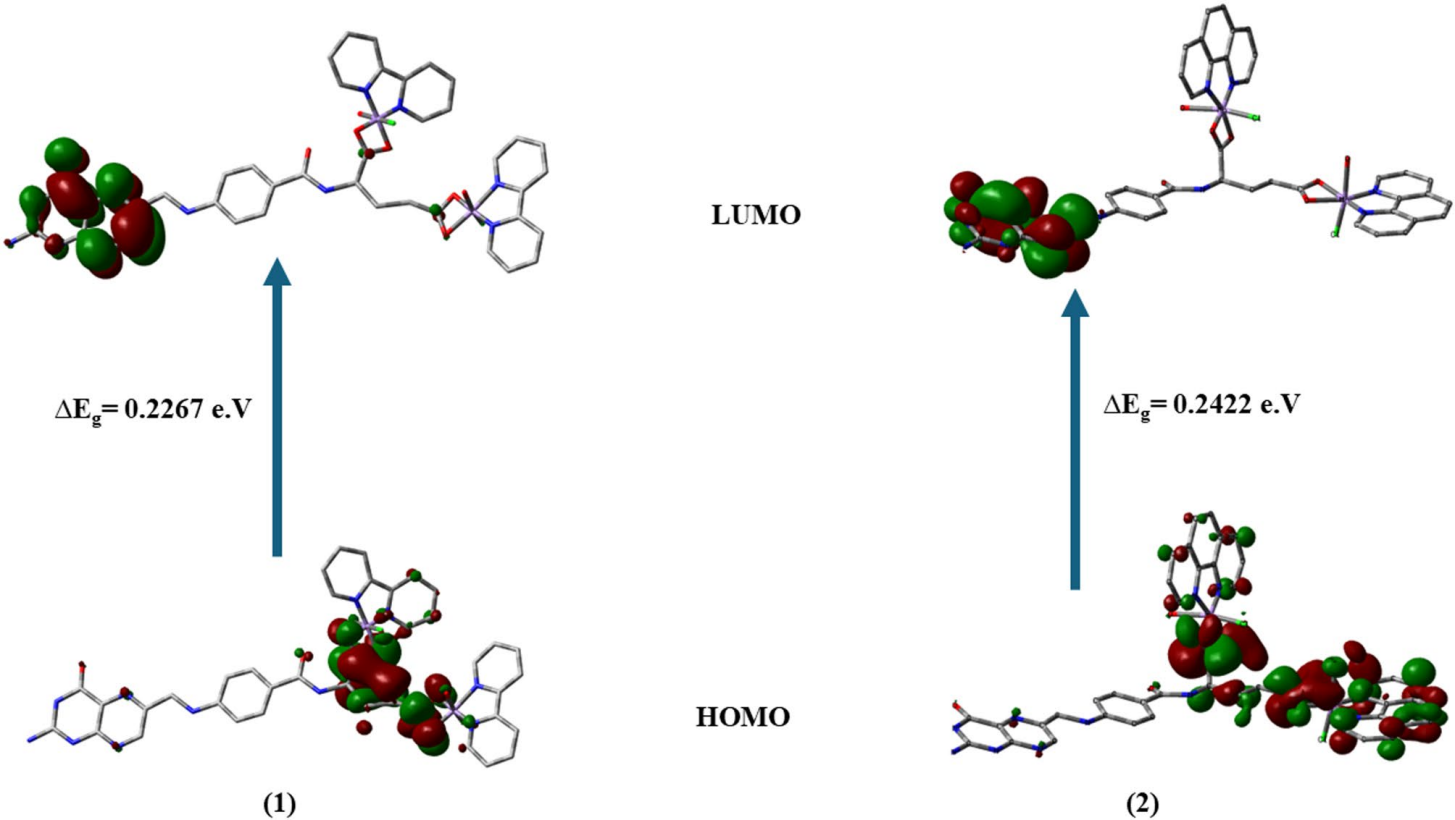
HOMO and LUMO charge density plots of the examined **Chelate 1** (**1**) and **Chelate 2** (**2**) utilizing B3LYP/6–311G++(d, p)-LANL2DZ level.

The ground state geometries of Chelate 1 and Chelate 2 are optimized using the mixed two basis sets B3LYP/6–311 + + g(d, p)-LANL2DZ. Fig. 5 offers the most stable tuned geometries in the gas phase, the vector of the dipole moment, and the atomic numbering arrangement of both chelates. Table 1 demonstrates the bond lengths and bond angles of the coordinating groups involved in chelation between each core metal ion (Mn1 and Mn2) and the different donor locations of FA, Bpy or Phen, H_2_O, and Cl ligands for both chelates. The coordination sphere around each Mn(II) obeys a deformed octahedral geometry. The atoms (N8, N9, O1, and O2) for Mn1 are almost in one plane, deviated by + 0.587° and − 5.012° for the Chelate 1 and Chelate 2, respectively. The atoms (N11, N10, O5, and O6) for Mn2 are almost in one plane, deviated by + 0.373° and + 0.068° for the Chelates 1 and 2, respectively. It was discovered from Table 2 that the energy of Chelate 1 is higher than Chelate 2 by 84.55 a.u., which reveals the higher stability of Chelate 2. Moreover, the computed E_HOMO_ and E_LUMO_ values and their energy gap (E_g_) were similar. The small value of E_g_ for the chelates indicates their high reactivity. The global reactivity descriptors, including the electronegativity (χ), absolute hardness (η), chemical potential (µ), absolute softness (σ), and global electrophilicity (ω), were estimated using Koopman’s approximation^63^. The values of these descriptors imply that both chelates demonstrate high global softness (S) values with a low degree of chemical hardness (η), and therefore, the charge transfer occurs more feasible. The total information from Table 2 indicates that Chelate 1 is less stable, showing higher reactivity and electron affinity than Chelate 2. Fig. 6 depicts the LUMOs and HOMOs for both complexes, the LUMOs are mainly distributed over the pteridine ring while the HOMOs are largely distributed over the glutamic acid of folate ligand with small participation from the co-ligands.

**Table 1:** Important optimized bond lengths (Å) and bond angles (°) of the [Mn_2_ (FA)(Bpy)_2_(H_2_O)_2_Cl_2_] (**Chelate 1**), and [Mn_2_ (FA)(Phen)_2_(H_2_O)_2_Cl_2_] **(Chelate 2**).

	Chelate 1	Chelate 2
Bond lengths (Å)		
Mn1-N8	1.201	1.489
Mn1-N9	1.510	1.730
Mn1-O1	1.210	1.249
Mn1-O2	1.526	1.971
Mn1-O7	1.914	2.130
Mn1-Cl1	2.051	1.964
Mn2-N10	1.261	1.712
Mn2-N11	1.742	1.713
Mn2-O5	1.710	1.942
Mn2-O6	1.231	1.904
Mn2-O8	1.924	2.149
Mn2-Cl2	2.024	1.891
Angles (°)		
N8-Mn1-N9	89.661	91.396
O1-Mn1-O2	86.450	80.626
O1-Mn1-N9	90.863	88.530
O2-Mn1-N8	92.993	98.893
O1-Mn1-Cl1	89.627	86.589
O2-Mn1-Cl1	90.506	91.057
N8-Mn1-Cl1	89.263	97.465
N9-Mn1-Cl1	87.880	95.716
O7-Mn1-N8	94.760	91.521
O7-Mn1-N9	88.213	88.352
O1-Mn1-N8	178.754	175.93
O2-Mn1-N9	176.876	166.857
O7-Mn1-Cl1	174.375	170.025
N10-Mn2-N11	86.401	89.747
O5-Mn2-O6	85.062	69.427
O5-Mn2-N10	98.552	101.158
O6-Mn2-N11	89.974	98.701
O5-Mn2-Cl2	93.528	87.513
O6-Mn2-Cl2	89.120	88.635
N10-Mn2-Cl2	91.347	97.903
N11-Mn2-Cl2	87.279	99.113
O8-Mn2-N10	93.081	88.823
O8-Mn2-N11	85.149	87.186
O8-Mn2-O5	93.592	84.965
O8-Mn2-O6	85.964	83.709
O5-Mn2-N11	174.955	166.403
O6-Mn2-N10	176.319	168.407
O8-Mn2-Cl2	170.968	170.762
N8- N9- O1-O2	+ 0.587*	-5.012*
N11-N10- O5-O6	+ 0.373*	+ 0.068*

*Dihedral angle.

**Table 2:** Calculated energies and properties of **Chelate 1** and **Chelate 2**.

Property	Chelate 1	Chelate 2
E (a.u.)	-4576.2627	-4660.8134
HOMO (eV)	-2.7982	-2.4988
LUMO (eV)	-2.57150	-2.3372
E_g_ (eV)	0.2267	0.2422
Dipole moment (Debye)	13.3353	9.7929
I=-E_HOMO_	2.7982	2.4988
A=-E_LUMO_	2.5715	2.3372
χ= (I + A)/2	2.6848	2.4180
η= (I -A)/2	0.1134	0.0808
S = 1/2η	4.411	6.1881
µ= -χ	-2.6849	-2.418
ω = µ^2^/2η	31.7972	36.1802

### DNA interaction assays

#### DNA binding

**Fig. 7:**
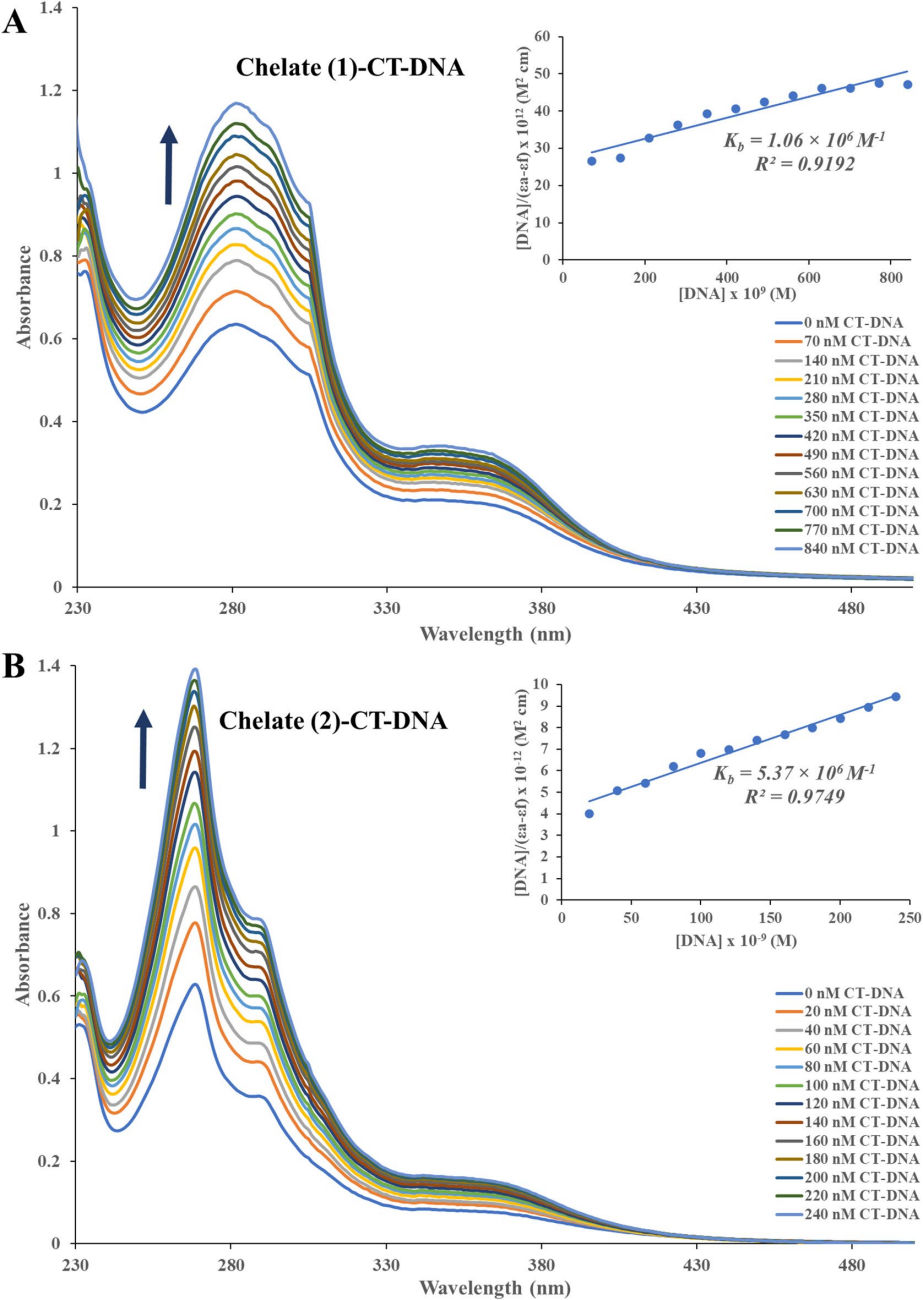
The absorbance spectrum of **Chelate 1** (**A**) and **Chelate 2** (**B**), in the absence and presence of rising concentrations of CT–DNA (0–840 nM) and (0–240 nM), respectively, accompanied by a marked hyperchromic shift, as indicated by the upward arrow.

The binding affinity of Chelate 1 and Chelate 2 to the CT-DNA was examined by electronic absorption spectroscopy. The binding of metal ions to nucleobase residues is influenced by the type of nitrogenous base: Hg^2+^ exhibits a high affinity for DNA regions rich in adenine (A) and thymine (T), whereas Cu^2+^, Mn^2+^, and Pt^2+^ preferentially bind to regions enriched in guanine (G) and cytosine (C)^64^. DNA binding is known to be mediated in several ways. Both covalent and non-covalent interactions are involved. An intra/interstrand cross-link is a representation of the irreversible binding that occurs during a covalent contact or metal-base coordination. Intercalation between DNA base pairs, insertion, major or minor groove binding, and electrostatic interactions with the sugar phosphate DNA backbone are examples of reversible binding that is typically a non-covalent interaction^65^. In cancer treatment, assessing the metal-based anticancer therapies’ affinities for DNA binding may be the first step toward understanding their mechanism of antitumor activity^44,66,67^.The absorption spectra associated with the binding interaction of the Chelates 1 and 2 with CT-DNA in the absence and presence of rising concentration of CT-DNA are presented in Fig. 7A,B, respectively. DNA binding interactions were analyzed using electronic absorption spectroscopy by observing changes in absorbance and wavelength shifts to assess binding characteristics^68^. The variation in DNA absorbance in the presence of the prepared chelates can serve as an indicator of interaction^69^. Our Chelates 1 and 2 exhibited a hyperchromic shift as the concentration of CT-DNA rises. The intrinsic binding constants (K_b_) were derived by plotting [DNA]/ (ε_a_ - ε_f_) against [DNA]. The values of K_b_ were calculated from the slope-to-intercept ratio of the linear graph, resulting in K_b_ values of 1.06 × 10^6^ M^−1^ for Chelate 1 and 5.37 × 10^6^ M^−1^ for Chelate 2. The binding constants of our binuclear chelates, reaching up to 10^6^ M^−1^, are comparable to those of classical DNA intercalators such as ethidium bromide and doxorubicin, with binding affinities in the range 10^5^–10^6^ M^-1^^70^. This suggests that the combination of Bpy/Phen intercalation with folate-mediated-hydrogen bonding and electrostatic interactions significantly enhances DNA binding strength, placing our chelates among the stronger DNA binders reported in the literature. The spectral changes suggest that the chelates interact strongly with DNA through non-covalent interactions, potentially causing the DNA double helix to unwind and exposing more DNA bases^71^. The two chelates, Chelate 1 and Chelate 2, interact with CT-DNA through non-covalent binding, primarily involving the nitrogen and oxygen atoms of the chelates and the base pairs in the minor grooves of the DNA. In addition to these interactions, van der Waals forces may also contribute to facilitating this binding. The type of interaction between the chelates and the CT-DNA helix was assessed by monitoring hyperchromic or hypochromic shifts. Hyperchromism indicates a change in the DNA structure and conformation following the chelate binding, which can lead to structural damage to the DNA helix^72–74^. However, in our study, this multifaceted interaction leads to significantly higher binding constants exceeding those typically reported for mononuclear Cu(II) or Mn(II)–Bpy/Phen complexes (10^3^–10^4^ M^−1^)^75,76^and even surpass some more elaborate Mn(II) systems incorporating sulfonated ligands (10^5^ M^−1^)^42^. These findings highlight the synergistic effect of the folate bridging ligand in combination with Bpy/Phen intercalation, resulting in stronger and more selective DNA binding than is typically observed in related mononuclear systems.

#### DNA cleavage

**Fig. 8:**
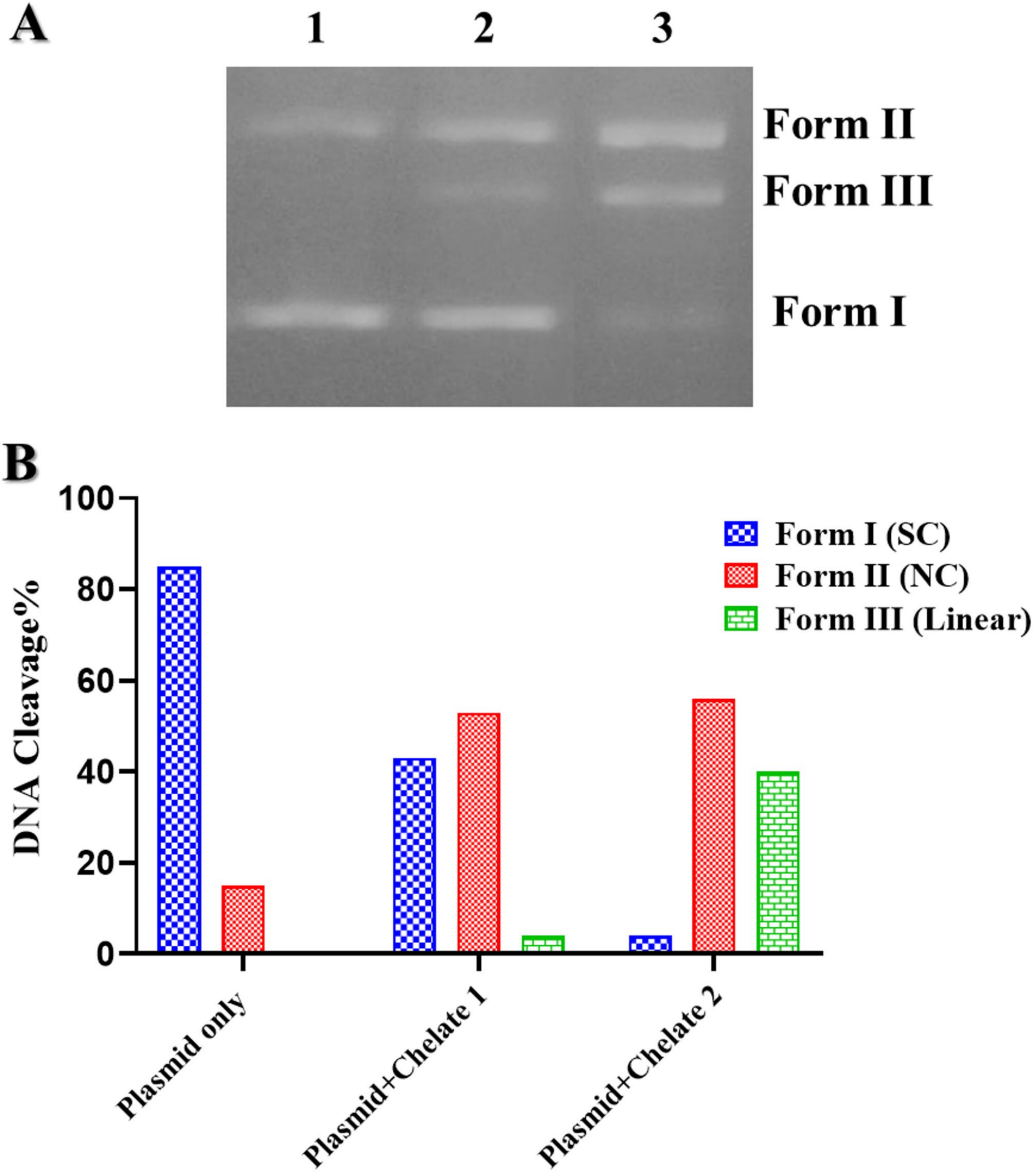
(**A**) The gel electrophoresis patterns show the cleavage of pBR322 plasmid DNA (~ 0.4 µg) by 100 µM of **Chelate 1** and **Chelate 2**, displayed in lanes 2 and 3, respectively, compared to lane 1, which represents the plasmid alone. (**B**) Bar representation diagram of the pBR322 plasmid DNA cleavage in the presence of Chelates **1** and **2**. The original gel image is presented in Fig. S1.

DNA is a key pharmacological target for many antitumor agents. Although eukaryotic DNA features a unique nucleosome structure compared to plasmid DNA, plasmid DNA is commonly utilized in research to draw connections between *in vitro* DNA damage and the biological responses of eukaryotic cells^77^. The nuclease activity of Chelate 1 and Chelate 2 was investigated under physiological pH and temperature conditions. In the presence of these metal chelates, plasmid DNA - initially in its supercoiled (SC), covalently closed circular form - can undergo cleavage. This cleavage results in either a nicked circular (relaxed) form due to single-strand breaks or a linear form caused by double-strand breaks^78,79^. The formation of nicked and linear forms of plasmid DNA, as observed in gel electrophoresis, is commonly linked to the interactions between DNA and metal chelates. The cleavage activity of Chelate 1 and Chelate 2 was assessed by tracking the gradual breakdown of intact supercoiled DNA (SC, Form I). Supercoiled DNA, the fastest migrating form (Form I), is converted into an open circular form (Form II) upon single-strand cleavage, resulting in reduced migration. In cases where both strands are cleaved, the DNA adopts a linear form (Form III), exhibiting an intermediate migration rate between Forms I and II^80^. The experimental results are shown in Fig. 8, where lane 1 represents the control sample of pBR322 plasmid DNA without any chelate treatment, showing no changes and remaining in its supercoiled form. Lane 2 corresponds to the plasmid DNA treated with Chelate 1, where the conversion of the supercoiled (SC, Form I) into the nicked circular (NC, Form II) is observed. Lane 3 corresponds to the plasmid treated with Chelate 2, which demonstrates the conversion of the SC form (Form I) into the nicked circular form (NC, Forms II) and linear form (Form III). These results indicate that both Chelate 1 and Chelate 2 exhibit DNA cleavage activity, but with varying efficiencies. Notably, Chelate 2 shows significantly higher DNA cleavage capability compared to Chelate 1. This observation is consistent with DNA binding studies, which reveal a higher binding constant for Chelate 2, further supporting its superior cleavage efficiency.

### Biological assays

#### *In vitro* cytotoxicity assay

Chelate 1 and Chelate 2 were tested for their cytotoxicity against both FR-positive and FR-negative cell lines. It is well established that the cellular uptake of folic acid-containing compounds is significantly higher in the FR-positive cell lines due to receptor-mediated endocytosis. FRs are valuable targets for tumor-specific delivery and receptor-mediated endocytosis of folic acid-containing compounds, as their expression is typically low or absent in healthy cells but significantly overexpressed in several human carcinomas, including those of the brain, breast, colon, and kidney^2,36,81–83^.

The cytotoxic behavior of a single dose (100 µg/ml) of the prepared chelates was investigated on the normal human skin fibroblasts (HSF), revealing an inhibition percentage of 4.7% for Chelate 1 and 8.5% for Chelate 2. This suggests that the synthesized chelates are relatively safe for the tested normal cells, compared to their higher cytotoxic effects on the tested cancer cells, indicating potential selective toxicity, as illustrated in Table 3.

**Table 3:** The Inhibition percentage of 100 µg/ml single dose of the prepared chelates, shows the anticancer effect of the prepared chelates on HCT116, hela, Caco-2, MCF-7, and A549 cell lines, the data were expressed as the mean ± SD of three independent experiments.

Cells	Inhibition percentage (%)	
	Chelate 1	Chelate 2
HCT116	79.3 ± 3.6	77.36 ± 0.55
HeLa	76.23 ± 1.1	75.03 ± 3.12
Caco-2	74.9 ± 2.33	74.49 ± 3.50
MCF-7	63.33 ± 5.11	60.84 ± 2.05
A549	57.9 ± 3.30	59.01 ± 3.02
HSF	4.7 ± 2.11	8.5 ± 1.71

**Fig. 9:**
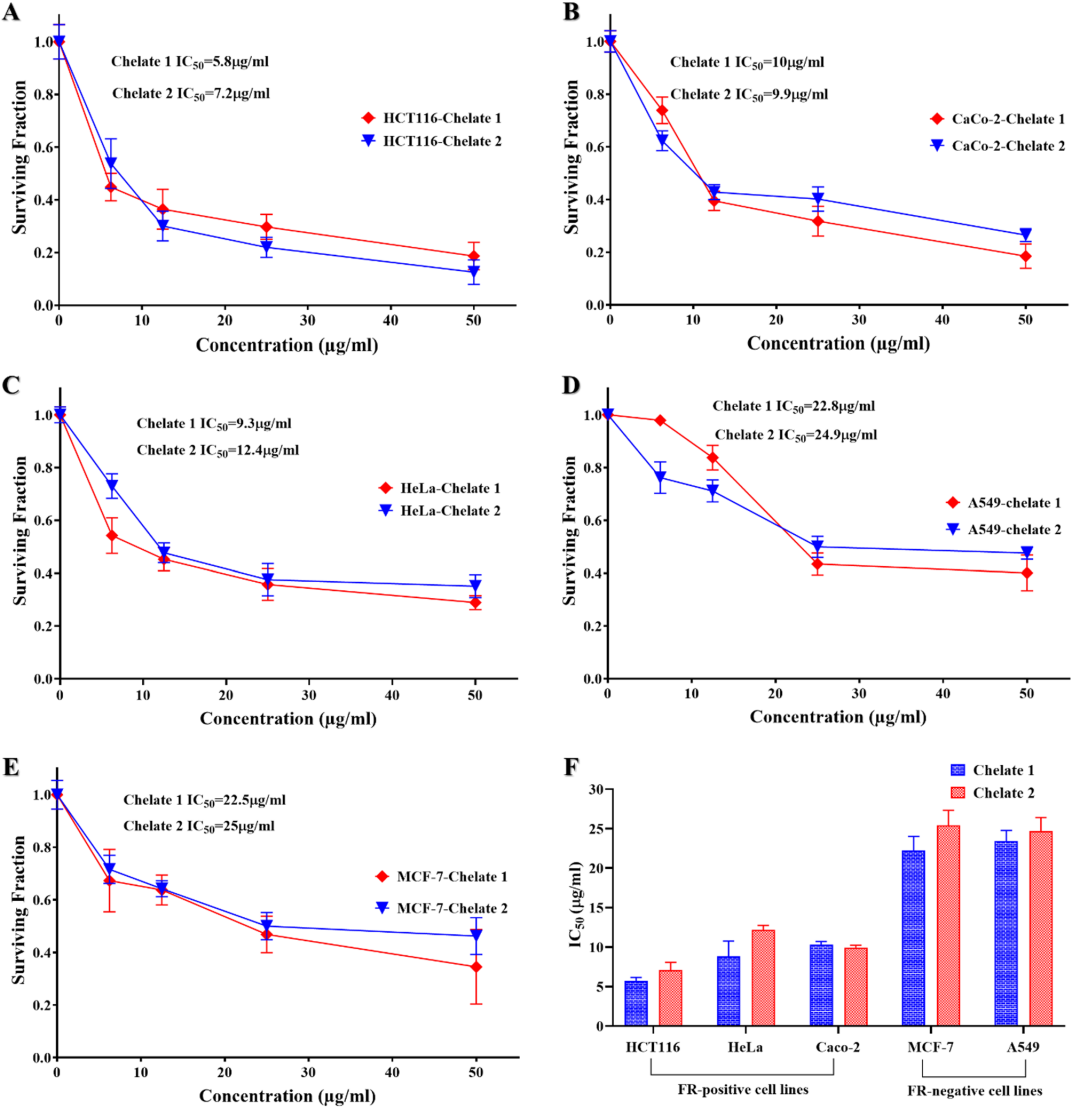
Cytotoxic activities of Chelates 1 and 2 revealed their effects on folate receptor-positive cell lines (HCT116, **A**; Caco-2, **B**; and HeLa, **C**) and folate receptor-negative cell lines (A549, **D**; and MCF-7, **E**), following 48 h of treatment. Chelates 1 and 2 demonstrated significant cytotoxic effects, as evidenced by their low IC_50_ values on cell lines that overexpress folate receptors compared to those lacking folate receptor expression (**F**).

The anticancer potential and IC_50_ values of the chelates were investigated on the basis of the folate receptor expression in the cancer cell lines. Regarding their cytotoxic effects on folate receptor-positive and folate receptor-negative cell lines, the chelates demonstrated significantly higher cytotoxicity toward folate receptor-positive cell lines compared to those lacking folate receptors, as illustrated in Table 4. The existence of folate receptors may have an impact on the chelates’ intake and anticancer action, which could provide credence to the idea of a receptor-mediated internalization process. BecauseFRs are overexpressed in several cancers, they are a prospective candidate for chemotherapy because they are essential for the selective absorption of folate and folate-conjugated compounds. The chelates exhibited a significantly strong anticancer effect on HCT116, HeLa, and Caco-2 cells, which are known for their high folate receptor expression on their surfaces^84–86^. On the other hand, MCF-7 and A549 cells, which express very little or no folate receptor^84,86,87^showed noticeably less cytotoxicity. Among the folate receptor-positive cell lines, the HCT116 cell line demonstrates the highest sensitivity towards the synthesized chelates with IC_50_ values of 5.8 ± 0.45 µg/ml for Chelate 1 and 7.2 ± 1.01 µg/ml for Chelate 2 compared to other cell lines (Fig. 9). Based on their efficacy in FR-positive cells, receptor-mediated endocytosis likely led to the chelates’ internalization, resulting in several potential toxic effects to the cellular machinery. The fact that folate receptor-negative cells exhibit noticeably less cytotoxicity suggests that passive diffusion is not the main uptake way of these chelates. This supports the idea that their anticancer action depends, at least in part, on internalization mediated by folate receptors. These results revealed the potential of the synthesized chelates as targeted anticancer agents that enhance selectivity while reducing toxicity to healthy, receptor-deficient cells by taking advantage of the overexpression of the folate receptor in cancer cells.

**Table 4:** Cytotoxic activity of **Chelate 1** and **Chelate 2** against folate receptor-positive and negative cell lines. The data were expressed as the mean ± SD of three independent experiments.

Type of cells based on folate receptor (FR) expression	Cells	IC_50_ (µg/ml)	
		Chelate 1	Chelate 2
FR-Positive cells	HCT116	5.8 ± 0.45	7.2 ± 1.01
	HeLa	9.3 ± 1.94	12.4 ± 0.5
	Caco-2	9.9 ± 0.37	10 ± 0.31
FR-Negative cells	MCF-7	22.3 ± 1.75	24.3 ± 1.90
	A549	24.9 ± 1.33	22.8 ± 1.70

#### Colony formation

**Fig. 10:**
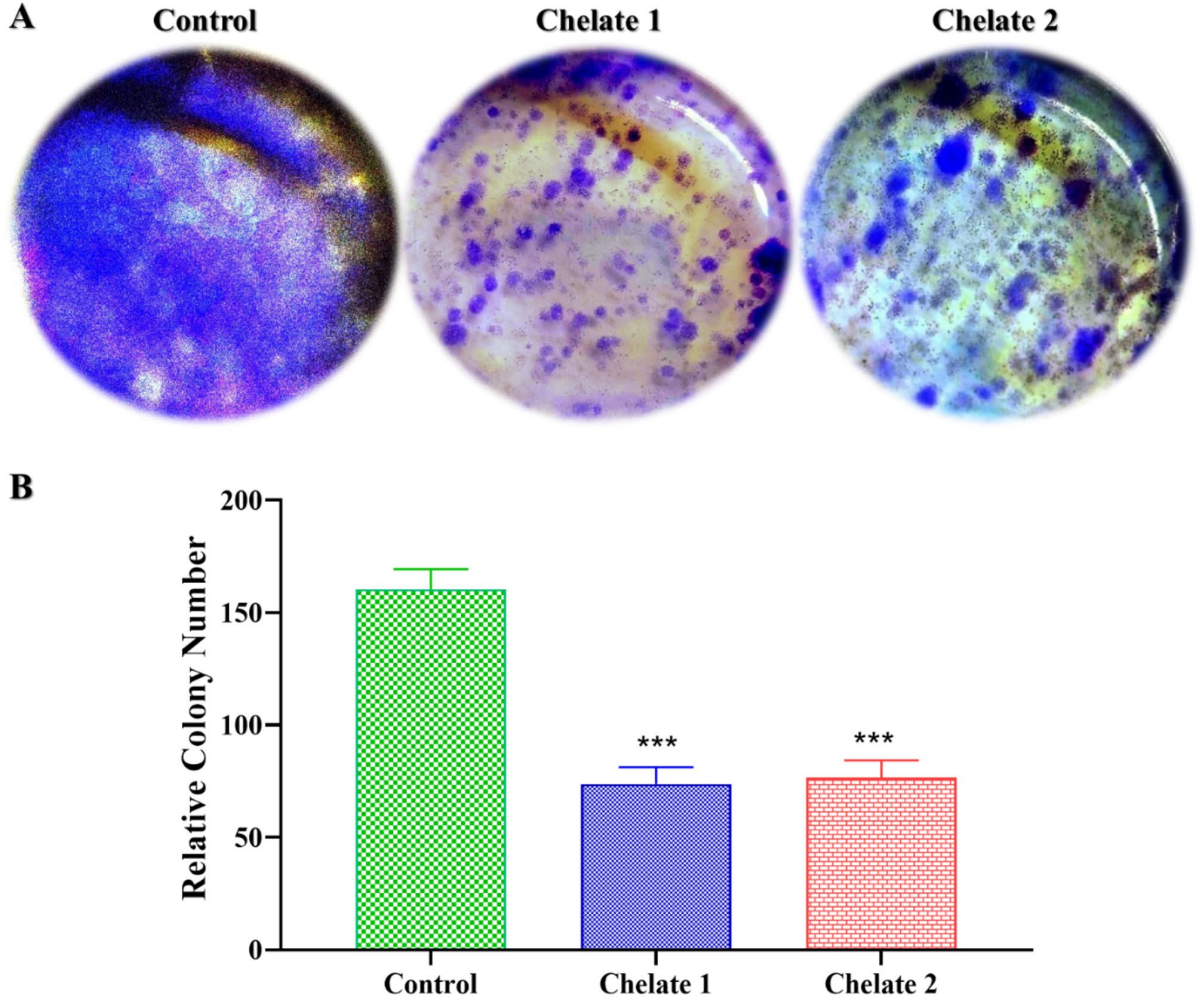
Suppressing of colony formation of HCT116 cells by the effect of **Chelate 1** and **Chelate 2**. (A) HCT116 cells were incubated without any treatment as a control, treated with the IC_50_ concentration of **Chelate 1**, and treated with the IC_50_ concentration of **Chelate 2**, then cultured in fresh medium for another 14 days, followed by staining with crystal violet. (B) The bar chart represents the decrease in the number of colonies after incubation with **Chelate 1** and **Chelate 2**. Data are represented as the mean ± SD of three independent experiments, ***P < 0.001 versus control. The statistical significance of the results was evaluated using a one-way ANOVA, followed by Tukey’s multiple comparison test to determine specific group differences.

A colony formation experiment was performed on HCT116 cells to examine further the prepared chelates’ potential as anticancer agents. This investigation assesses the capability of cancer cells to endure and form colonies after being exposed to cytotoxic substances, which offers important insights into the long-term proliferative potential of cancer cells after treatment with medications. Cancer cells proliferate in colonies in close proximity to other cells; when these connections are lost, the cancer cells eventually die^88^. HCT116 cells treated with the IC_50_ concentration of Chelate 1 and Chelate 2 for 48 h disclosed a considerably lower number of colonies compared to the untreated control group (Fig. 10). In particular, the Chelate 1 and Chelate 2 treatment groups produced 73.7 ± 7.6 and 76.7 ± 7.6 colonies, respectively, compared to the control group’s average of 160.3 ± 9 colonies. This noteworthy reduction in colony formation demonstrates the Chelates 1 and 2’s potent cytotoxic effects on HCT116 cells. This resilient suppression of colony formation suggests that the chelates mitigate long-term clonogenic survival, a key hallmark of aggressive cancer phenotypes, in addition to hindering short-term cell viability. These results demonstrate the capacity of Chelate 1 and Chelate 2 to impede the growth of cancer cells and further support their anti-proliferative capability.

#### Wound healing

**Fig. 11:**
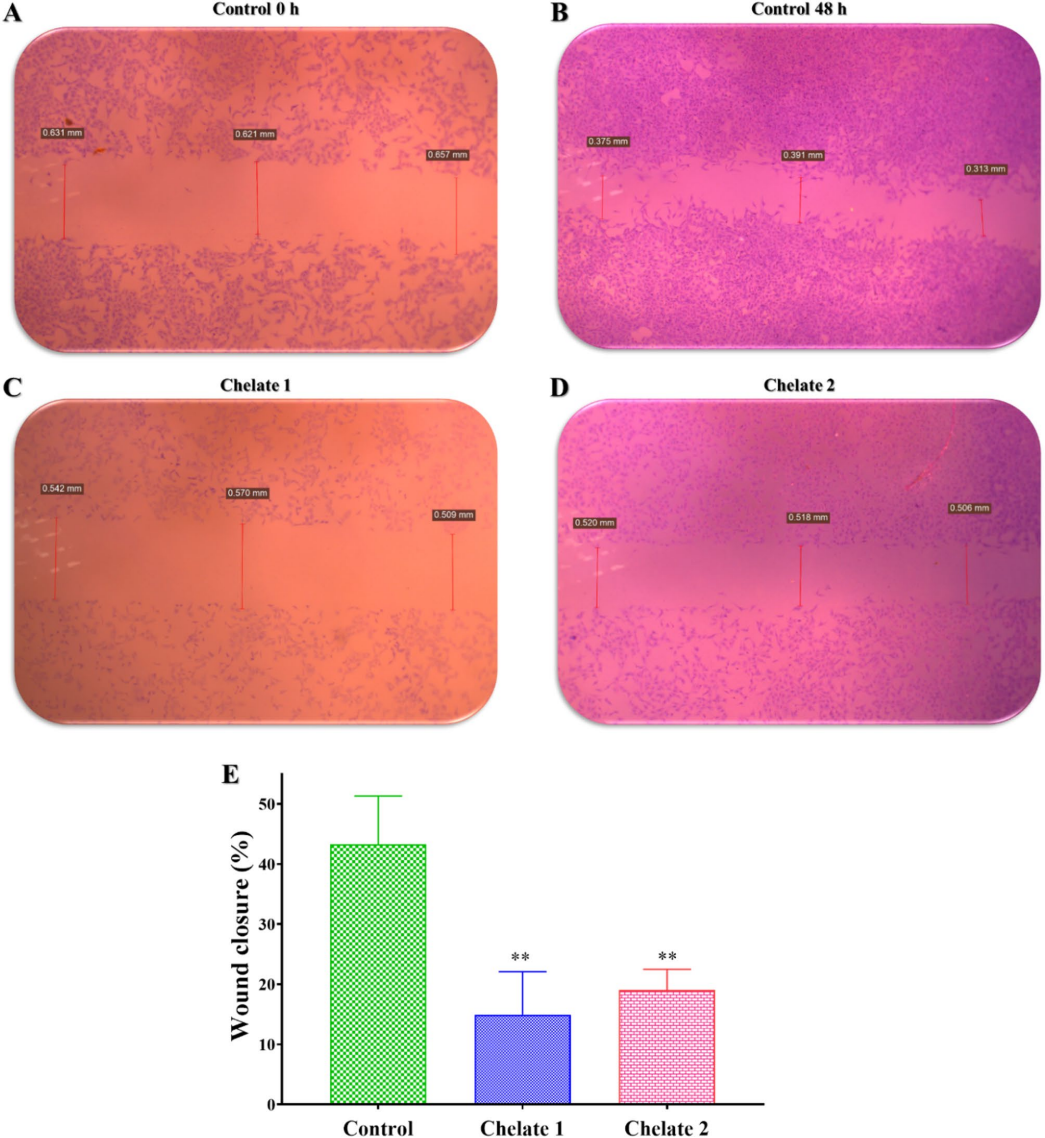
The inhibitory effect of the prepared chelates on the migration of HCT116 cells that was treated by their IC_50_ concentrations and compared to the untreated HCT116 cells (control group). (**A–D**) Images show changes in wound size after treatment with **Chelate 1 and Chelate 2** for 48 h, as compared to control. (**E**) Bar chart illustrates the percentage of wound closure, data are represented as the mean ± SD of three independent experiments, **P < 0.01 means statistically significant from the control. The statistical significance of the results was evaluated using a one-way ANOVA, followed by Tukey’s multiple comparison test to determine specific group differences.

To estimate the migration and proliferation profile of the tested chelates, a scratch wound assay was performed on HCT116 cells, by creating an artificial wound gap and then monitoring the closure of this gap by the mean of cell migration and proliferation^50^. The synthesized Chelate 1 and Chelate 2 significantly impaired the migration capabilities and wound closure of HCT116 cells compared to the control group (Fig. 11). Particularly, after 48 h, the groups treated with Chelate 1 and Chelate 2 had substantially lower wound closure rates of 14.9% ± 7.2 and 19.1% ± 3.4, respectively, compared to the untreated control group’s average of 43.3% ± 8.0. These results suggested that Chelate 1 and Chelate 2 can suppress the cell migration of HCT116. This study, alongside the colony-forming assay, reveals that the synthesized chelates inhibit two key processes required for cancer cell growth and spread: colony formation and migration, both crucial for metastasis. The chelates effectively hinder these essential activities in HCT116 cells. Metastasis in cancer involves intricate biological processes, such as invasion and migration, and remains a leading cause of cancer-related deaths^89^.

#### Cell morphology

**Fig. 12:**
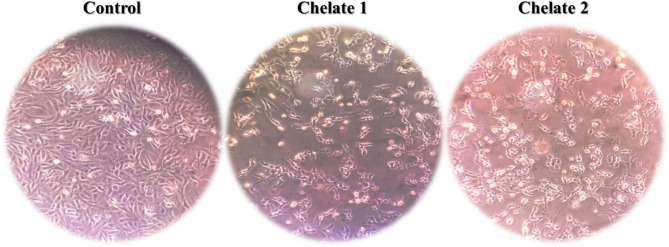
Morphological changes in the HCT116 cells following treatment with the prepared chelates, compared to the untreated control, as observed under an inverted microscope.

HCT116 cells treated separately with Chelate 1 and Chelate 2 displayed distinct morphological changes as shown in Fig. 12. These included fragmentation of the nuclei, damage to the membrane of the cells, cell shrinkage, and declination in cell size, all indicative of programmed cell death^90,91^. This will be further confirmed by evaluating the expression of apoptotic and anti-apoptotic proteins, specifically Bax, BCL-2, and Caspase-3, which serve as key markers for apoptosis pathways. These findings reinforce the chelates’ role in inducing apoptosis within treated cells, as shown by both morphological observations and protein expression analysis.

#### Cell cycle

**Fig. 13:**
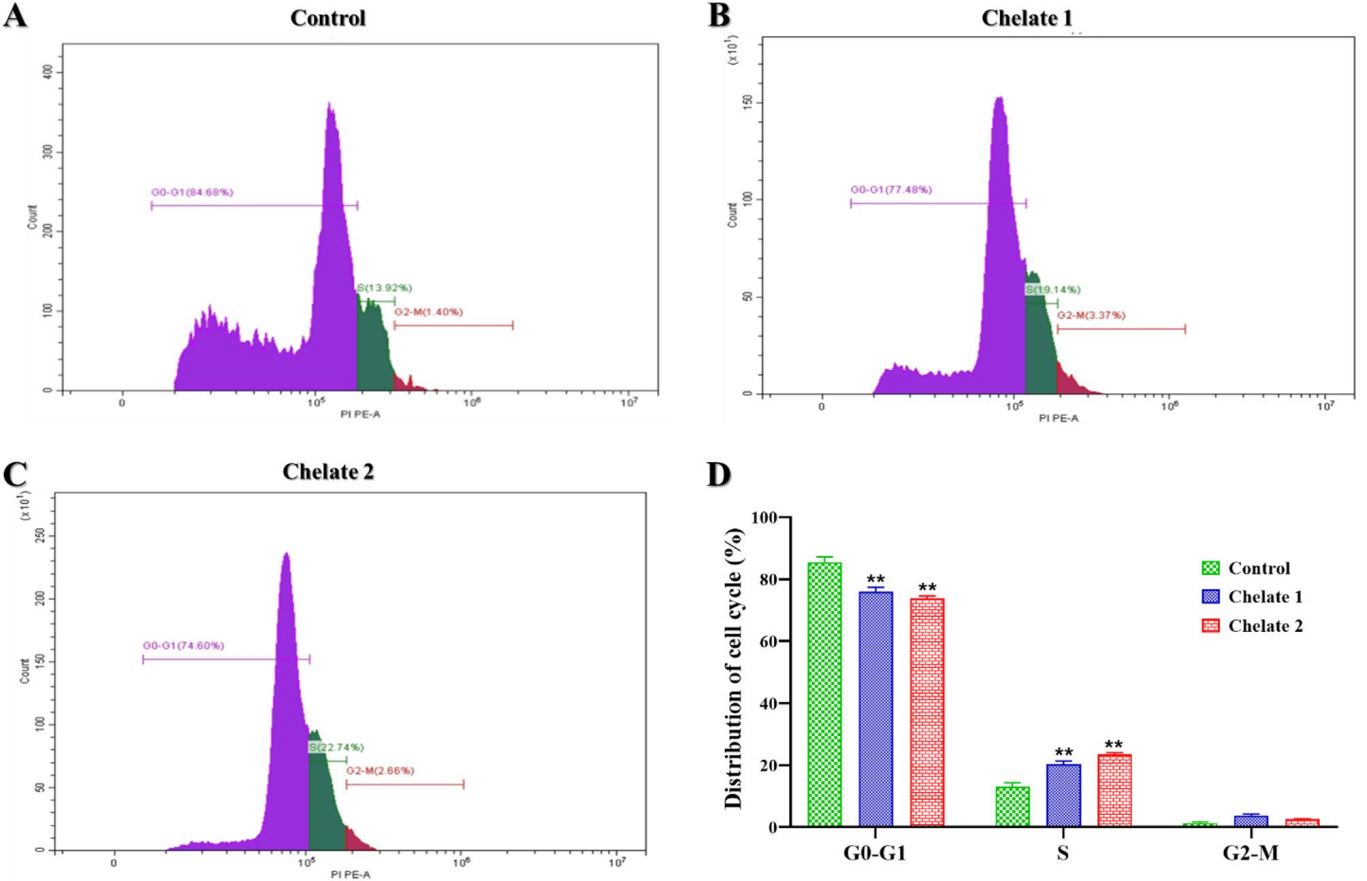
The distribution of the cell cycle of (**A**) untreated HCT116 cells, (**B**) HCT116 cells treated with **Chelate 1,** and (**C**) HCT116 cells treated with **Chelate 2**, as measured by flow cytometry. (**D**) Bar chart represents a significant decrease in the proportion of cells in the G0-G1 phase, while a significant accumulation of cells is observed in the S-phase. Data are represented as the mean ± SD of three independent experiments, **P < 0.01 means statistically significant from the control. The statistical significance of the results was evaluated using a one-way ANOVA, followed by Tukey’s multiple comparison test to determine specific group differences.

The cell cycle serves as the fundamental process underlying cell proliferation^92–95^. To assess the effects of Chelate 1 and Chelate 2 on the cell cycle progression of the HCT116 cell line, cell cycle distribution was evaluated using flow cytometry. Cells were treated with the IC_50_ concentration of each chelate separately. As presented in Fig. 13, treatment with Chelate 1 (5.8 µg/mL) and Chelate 2 (7.2 µg/mL) for 48 h led to a significant accumulation of cells in the S phase. Specifically, the S phase population increased from 13.1% ± 1.2 in the control group to 20.3% ± 1.0 in Chelate 1-treated cells and 23.4% ± 0.6 in Chelate 2-treated cells. Correspondingly, the percentage of cells in the G0/G1 phase decreased from 85.6% ± 1.6 in the control to 76.0% ± 1.4 and 73.9% ± 0.7 in cells treated with Chelate 1 and Chelate 2, respectively. A slight increase in G2/M phase was also observed, rising from 1.3% ± 0.4 in the control to 3.7% ± 0.5 with Chelate 1, and 2.7% ± 0.02 with Chelate 2. These findings suggest that the chelates reduce cell proliferation by inducing S-phase arrest in the cell cycle. The cell cycle is divided into distinct phases, each characterized by specific activities. The S phase, occurring before cell division, is marked by DNA and histone synthesis, essential for preparing the cell’s genetic material for distribution to daughter cells during division^96–98^. In case of fragmentation of DNA which may occurred during replication, replication will be halted and the mechanism for intracellular DNA repair will start at the same time to enable the continuation of the DNA replication process. At the S phase checkpoint, the phosphorylation of the CDK2/Cyclin E complex plays a critical role in this repair process by extending the duration of the S phase, thereby providing sufficient time for repair before replication continues. Our results indicate that Chelate 1 and Chelate 2 caused the accumulation of cells in the S phase. These data would also be further confirmed by investigating the expression of the protein levels of CDK2 and Cyclin E^98,99^.

#### Enzyme-linked immunosorbent assay

**Fig. 14:**
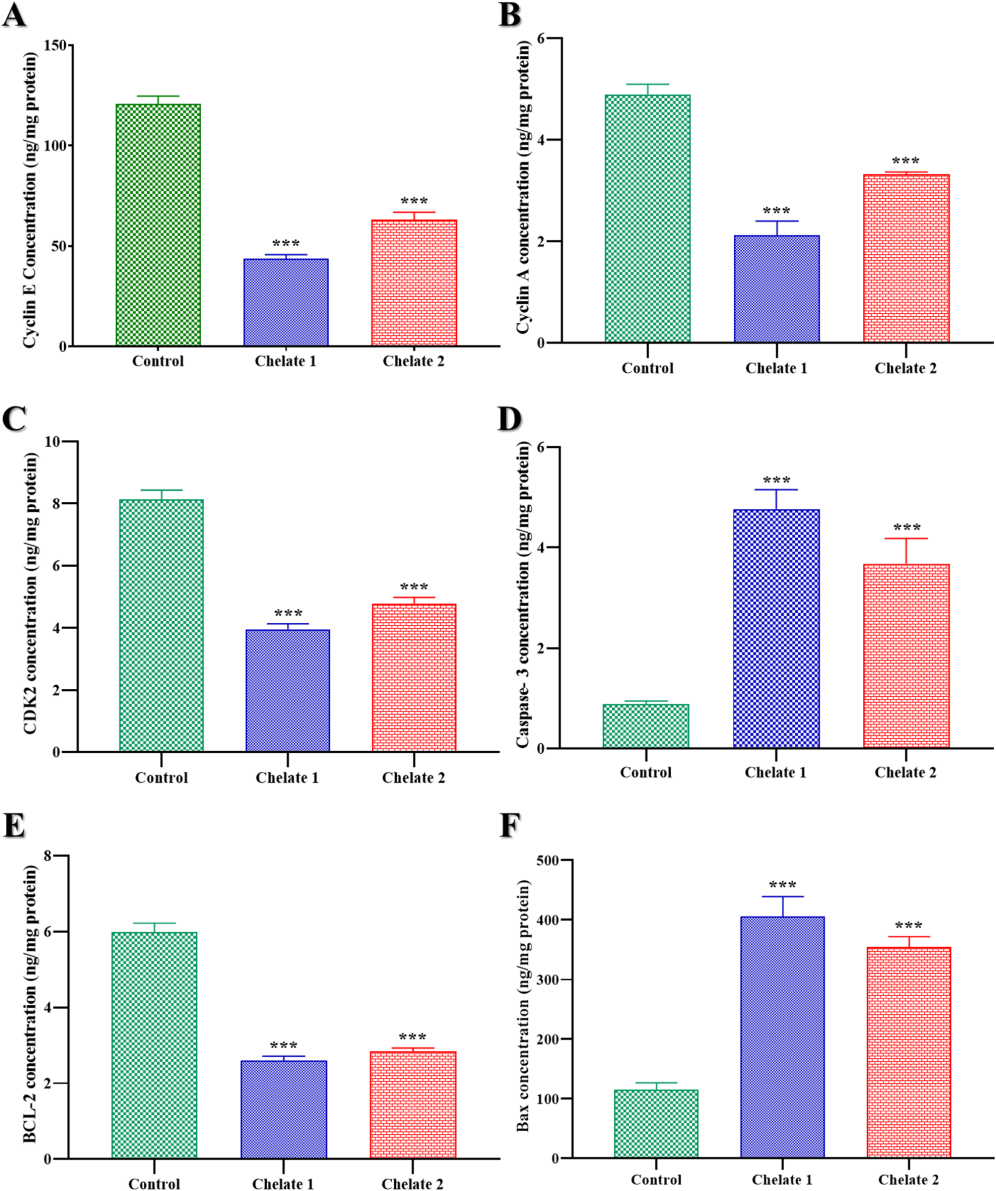
Evaluation of protein expression levels using ELISA analysis for Cyclin E (**A**), Cyclin A (**B**), CDK2 (**C**), caspase-3 (**D**), BCL-2 (**E**) and Bax (**F**) proteins in HCT116 cells treated with IC_50_ concentration of **Chelate 1** and **Chelate 2** compared to the untreated HCT116 which act as control group. Data were obtained as mean ± SD of three independent experiments, ***P < 0.001 means statistically significant from control. The statistical significance of the results was evaluated using a one-way ANOVA, followed by Tukey’s multiple comparison test to determine specific group differences.

In order to evaluate the effects of Chelate 1 and Chelate 2 on colorectal cancer cells (HCT116), we assessed the levels of key oncogenic and apoptotic proteins using ELISA. Cyclin E, Cyclin A, and CDK2 are critical regulators of cell cycle progression and are frequently overexpressed in cancer. Meanwhile, Caspase-3, Bax, and Bcl-2 are pivotal in the regulation of apoptosis.  The HCT116 cells treated with the IC_50_ of each Chelate 1 and Chelate 2 show a significant increase in the expression of caspase-3 and Bax proteins while considerable decline in the expression of Cyclin E, Cyclin A, CDK2 and Bcl-2 compared to the control, as represented in Fig. 14. In particular, Cyclin E levels decreased from 120.72 ± 3.94 ng/mg protein in control cells to 43.59 ± 2.17 ng/mg (fold change ≈ 0.36) and 63.18 ± 3.69 ng/mg (fold change ≈ 0.52) in Chelate 1- and Chelate 2-treated cells, respectively. Similarly, Cyclin A levels decreased from 4.89 ± 0.20 ng/mg protein in control cells to 2.12 ± 0.28 ng/mg (fold change ≈ 0.43) and 3.32 ± 0.04 ng/mg (fold change ≈ 0.68) in Chelate 1- and Chelate 2-treated cells, respectively. Likewise, CDK2 levels were reduced from an average of 8.12 ± 0.30 ng/mg in control cells to 3.95 ± 0.18 ng/mg (fold change ≈ 0.49) in Chelate 1-treated cells and 4.78 ± 0.19 ng/mg (fold change ≈ 0.59) in Chelate 2-treated cells. In contrast, Caspase-3 expression increased markedly from 0.88 ± 0.06 ng/mg protein in control cells to 4.77 ± 0.38 ng/mg (fold change ≈ 5.4) with Chelate 1 and 3.68 ± 0.47 ng/mg (fold change ≈ 4.2) with Chelate 2. Conversely, Bcl-2 levels decreased from 5.98 ± 0.24 ng/mg protein in control cells to 2.60 ± 0.11 ng/mg (fold change ≈ 0.43) with Chelate 1 and 2.84 ± 0.10 ng/mg (fold change ≈ 0.47) with Chelate 2. Bax expression significantly increased from 115.57 ± 10.63 ng/mg protein in control cells to 405.41 ± 33.73 ng/mg (fold change ≈ 3.5) with Chelate 1 and 354.58 ± 14.26 ng/mg (fold change ≈ 3.1) with Chelate 2. The progression of the cell cycle from the G1 phase to the S phase depends on cyclin E, a crucial regulator of cyclin-dependent kinases (CDKs). Cyclin-dependent kinase 2 (CDK2) and its regulatory partners, Cyclin E and Cyclin A, are principally responsible for the S phase transition and the start of DNA replication. Although both cyclins aid in DNA replication, Cyclin A sustains CDK2 activity to enable DNA synthesis throughout the S phase, whereas Cyclin E initially activates CDK2 at the G1/S checkpoint. This sequence demonstrates the distinct but related roles that Cyclin E and Cyclin A play in controlling the advancement of the cell cycle and the start of DNA replication^100–102^. Together with its companion CDK2, cyclin E and cyclin A are crucial for controlling the cell cycle, especially during the G1 phase transition to the S phase. These cyclins are overexpressed in many malignancies, including colorectal cancer, which causes unchecked cell growth. Because it pushes cells into the S phase too soon, causing genomic instability, a defining feature of cancer progression. Cyclin E, in particular, is regarded as an oncogene. In colorectal cancer, increased Cyclin E levels have been linked to tumor aggressiveness and a poor prognosis^103^. Cyclin A and CDK2 collaborate closely to control the S phase, and they are both linked to the growth of cancer cells. Uncontrolled cell division brought on by Cyclin A overexpression may promote the formation of tumors. The cell cycle is largely driven by CDK2, which forms complexes with both Cyclin E and Cyclin A. These cyclins’ carcinogenic qualities are reinforced by their hyperactivation in cancer cells, which makes them vital targets for cancer therapy^104^. The fact that these molecules are oncogenes makes their inhibition a promising strategy for cancer therapy. With regards to apoptosis, Bax and caspase-3 proteins are considered tumor suppressor proteins with pro-apoptotic functions^105^while Bcl-2 protein is a frequent overexpressed anti-apoptotic oncogene found in a variety of cancer types, by blocking the release of cytochrome c from mitochondria, a crucial step in activating caspases, the enzymes that cause cell death, it primarily prevents programmed cell death, this apoptosis resistance encourages the survival of cancer cells and the growth of tumors^106^. Caspase-3 is one essential apoptosis executor. When it is activated, important cellular components are cleaved, which ultimately results in programmed cell death. Higher levels of apoptosis in cancer cells are generally linked to increased caspase 3 activity, which is frequently the intended result when using anticancer medications to treat cancer. Changes in Bcl-2 and Bax levels further support this, as Bcl-2 suppresses apoptosis while Bax promotes it. In cancer cells, a higher Bax/Bcl-2 ratio is frequently linked to increased apoptotic activity^107^. Here, the Bax/Bcl-2 ratio increased to approximately 8.06 and 6.47 in Chelate 1- and Chelate 2-treated cells, respectively, indicating a strong apoptotic response. The prepared chelates can induce apoptosis in the colorectal cancer cell line (HCT116) and stop its progression by arresting the cell cycle at S-phase and reducing cancer cell proliferation. Relative protein expression (fold change/control was determined as shown in Table 5.

**Table 5:** ELISA for determination of relative protein expression (fold change/ control) in HCT116 cells treated with Chelates 1 and 2 (IC_50_).

Relative protein expression (fold change/control)		
Protein	Chelate 1/HCT116	Chelate 2/HCT116
Cyclin E	0.36112	0.523401
Cyclin A	0.433156	0.6788
CDK2	0.486642	0.58820
Caspase-3	5.390271	4.1617
BCL-2	0.435022	0.474159
Bax	3.5079	3.068

## Concluding remarks and future directions

This study presents exploratory *in vitro* evidence supporting the anticancer potential of two novel binuclear Mn(II) chelates, Chelate 1 and Chelate 2. They show significant binding affinity, particularly Chelate 2, which exhibited superior performance. This higher affinity translated into an enhanced ability of Chelate 2 to cleave plasmid DNA effectively. Additionally, both folate-based chelates displayed notable cytotoxicity against folate receptor-positive cell lines, especially HCT116, where their effects were pronounced. Conversely, their impact on folate receptor-negative cell lines was comparatively less significant. The potent effects of the synthesized chelates on HCT116 cells were further investigated through their ability to close gaps in a wound healing assay and their potent inhibitory activity against colony formation. Both chelates were found to arrest the cell cycle at the S-phase by downregulating the expression of Cyclin E, Cyclin A, and CDK2. Moreover, they increased the expression of pro-apoptotic proteins (Bax and Caspase 3) while reducing the levels of anti-apoptotic proteins (Bcl2), indicating their ability to induce apoptosis. This was further confirmed by the observed morphological changes in treated cells. Based on these findings, chelates may be able to cause death and stop the cell cycle from progressing to S-phase. Nevertheless, these findings are heavily dependent on *in vitro* data obtained from a single colorectal cancer cell line. We acknowledge that more *in vivo* research and testing across cancer models are necessary to confirm these results, but we have not exaggerated our results, even if the molecular mechanisms are interesting and fit known carcinogenic pathways. This work provides a preliminary foundation for future studies evaluating the therapeutic potential of metal chelates and recognizes the limits of exploratory *in vitro* research. To support any possible therapeutic uses, further studies should investigate toxicity and pharmacokinetics, confirm these effects in animal models, and dig deeper into the molecular processes at work.

## Electronic supplementary material

Below is the link to the electronic supplementary material.

Supplementary Material 1

## Data Availability

All data generated or analysed during this study are included in this published article (and its Supplementary Information files).
