# Supplementary material for: Folate-based binuclear Mn(II) chelates with 2,2’-bipyridine/1,10-phenanthroline as targeted anticancer agents for colon cancer cells
Source: Sci Rep. 2025 Jul 31;15:27905. doi: 10.1038/s41598-025-12251-9 (PMC12313882; doi:10.1038/s41598-025-12251-9)
Supplement: Supplementary file 1 — Supplementary Material 1 [file 41598_2025_12251_MOESM1_ESM.docx]

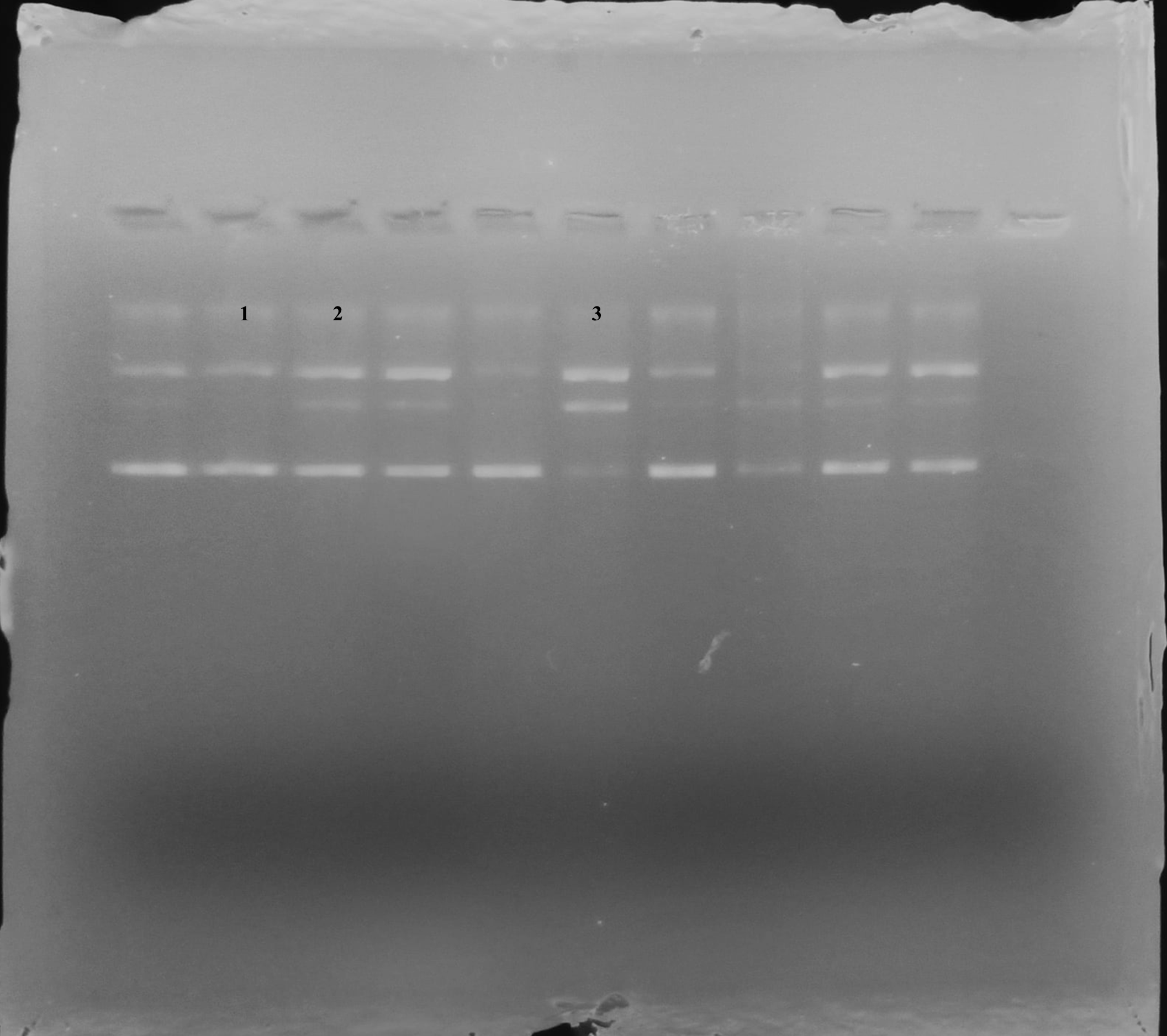


**Figure S1.** Uncropped gel image for the gel electrophoresis pattern shows the cleavage of pBR322 plasmid DNA (~0.4 µg) by 100 μM of chelates (1) and (2), displayed in lanes 2 and 3, respectively, compared to lane 1, which represents the plasmid alone.
